# Development and application of high-throughput screens for the discovery of compounds that disrupt ErbB4 signaling: Candidate cancer therapeutics

**DOI:** 10.1371/journal.pone.0243901

**Published:** 2020-12-30

**Authors:** Richard L. Cullum, Lauren M. Lucas, Jared I. Senfeld, John T. Piazza, Logan T. Neel, Kanupriya Whig, Ling Zhai, Mackenzie H. Harris, Cristina C. Rael, Darby C. Taylor, Laura J. Cook, David P. Kaufmann, Christopher P. Mill, Megan A. Jacobi, Forrest T. Smith, Mark Suto, Robert Bostwick, Ram B. Gupta, Allan E. David, David J. Riese, II

**Affiliations:** 1 Department of Drug Discovery and Development, Auburn University, Auburn, AL, United States of America; 2 Department of Chemical Engineering, Auburn University, Auburn, AL, United States of America; 3 Department of Biological Sciences, Auburn University, Auburn, AL, United States of America; 4 Drug Discovery Division, Southern Research, Birmingham, AL, United States of America; 5 Department of Chemistry and Biochemistry, Auburn University, Auburn, AL, United States of America; 6 Department of Chemical and Life Science Engineering, Virginia Commonwealth University, Richmond, VA, United States of America; 7 Department of Leukemia, Division of Cancer Medicine, University of Texas M.D. Anderson Cancer Center, Houston, TX, United States of America; Osmania University, INDIA

## Abstract

Whereas recent clinical studies report metastatic melanoma survival rates high as 30–50%, many tumors remain nonresponsive or become resistant to current therapeutic strategies. Analyses of The Cancer Genome Atlas (TCGA) skin cutaneous melanoma (SKCM) data set suggests that a significant fraction of melanomas potentially harbor gain-of-function mutations in the gene that encodes for the ErbB4 receptor tyrosine kinase. In this work, a drug discovery strategy was developed that is based on the observation that the Q43L mutant of the naturally occurring ErbB4 agonist Neuregulin-2beta (NRG2β) functions as a partial agonist at ErbB4. NRG2β/Q43L stimulates tyrosine phosphorylation, fails to stimulate ErbB4-dependent cell proliferation, and inhibits agonist-induced ErbB4-dependent cell proliferation. Compounds that exhibit these characteristics likely function as ErbB4 partial agonists, and as such hold promise as therapies for ErbB4-dependent melanomas. Consequently, three highly sensitive and reproducible (Z’ > 0.5) screening assays were developed and deployed for the identification of small-molecule ErbB4 partial agonists. Six compounds were identified that stimulate ErbB4 phosphorylation, fail to stimulate ErbB4-dependent cell proliferation, and appear to selectively inhibit ErbB4-dependent cell proliferation. Whereas further characterization is needed to evaluate the full therapeutic potential of these molecules, this drug discovery platform establishes reliable and scalable approaches for the discovery of ErbB4 inhibitors.

## 1. Introduction

In the last decade, the standard-of-care treatment options for patients with metastatic melanoma has significantly improved with the advent of targeted therapies and immunotherapies. In fact, recent studies with combination therapy using BRAF and MEK inhibitors and immunotherapies indicate rates of 5-year progression free survival and overall survival as high as 30–50% for metastatic melanoma patients whose tumors harbor a gain-of-function mutation in the *BRAF* gene [1–6]. However, there are still no effective targeted therapies for the treatment of melanomas that harbor the wild-type *BRAF* gene.

Our ongoing, unpublished analyses of the TCGA skin cutaneous melanoma genomic (SKCM) data set reveal that 70 of the 470 (15%) of the melanoma genomes harbor at least one non-synonymous missense mutation in the *ERBB4* gene. These 70 cases contain a total of 82 *ERBB4* missense mutant alleles. A total of 71 unique *ERBB4* missense mutant alleles are observed; the R711C mutant allele is observed in five cases; the R106C mutant allele is observed in three cases; the E452K, P759L, D813N, R992C, and S975L mutant alleles are observed in two cases; the remaining 64 mutant alleles are observed in only one case each. The degree of these coincident mutant alleles is greater than would be expected from a random distribution of 82 missense mutations across the *ERBB4* coding sequence (1308 amino acids), Similarly, the incidence of *ERBB4* mutations is higher among residues that are predicted to be important for ErbB4 function. Finally, the incidence of *ERBB4* mutations is higher in cases that contain a wild-type *BRAF* gene and is higher in cases that also harbor a mutation predicted to result in elevated RAS signaling. Taken together, these data indicate that *ERBB4* mutations appear to function as tumor drivers in *BRAF* wild-type melanomas by cooperating with elevated RAS signaling. Therefore, the discovery of strategies for inhibiting ErbB4 coupling to melanoma cell proliferation is a priority.

In multiple systems, ErbB4-dependent cellular proliferation requires ErbB4 heterodimerization with EGFR or ErbB2 and ErbB4 phosphorylation by EGFR or ErbB2 [7–13]. This suggests it may be possible to treat ErbB4-dependent melanomas using anti-EGFR and -ErbB2 therapies, particularly small molecule EGFR and/or ErbB2 tyrosine kinase inhibitors. Indeed, there are several FDA-approved EGFR and ErbB2 kinase inhibitors [14]. However, the responses to these therapies are often limited by the development of resistance mechanisms [15, 16]. Furthermore, targeting EGFR and ErbB2 for the treatment of ErbB4-dependent tumors could potentially affect the normal physiological roles of EGFR and ErbB2 in healthy tissue.

Consequently, we believe that compounds that function as partial agonists of ErbB4 hold great promise for the treatment of ErbB4-dependent melanomas. Our drug discovery strategy is driven by our observation that introducing the Q43L mutation into the gene that encodes the naturally-occurring ErbB4 full agonist NRG2β creates a partial agonist of ErbB4 [7]; this NRG2β/Q43L mutant stimulates ErbB4 tyrosine phosphorylation, yet it inhibits agonist-induced ErbB4-dependent cell proliferation. This antagonistic activity can be overcome with an excess of wild-type NRG2β (competitive inhibition) and appears to be the effect of phosphorylation-dependent downregulation of ErbB4.

Here we have developed a screening process for identifying compounds that function as partial agonists of ErbB4. Moreover, implementation of this process has yielded several compounds that appear to selectively disrupt ErbB4 signaling, and as such are predicted to possess therapeutic potential against ErbB4-dependent tumors.

## 2. Results

### 2.1. ErbB4 tyrosine phosphorylation can be stimulated and detected via semi-automated 96-well assays

#### 2.1.1. Development and validation of a 96-well sandwich ELISA for the detection of ErbB4 tyrosine phosphorylation

Our ultimate goal is the identification of ErbB4 partial agonists that inhibit coupling of ErbB4 to cell proliferation, preferably through stimulation of phosphorylation-dependent ErbB4 turnover and degradation. Thus, the first goal of these experiments is to develop and deploy an assay for stimulation of ErbB4 tyrosine phosphorylation. The CEM/ErbB4 cell line previously validated for assaying ligand-induced ErbB4 tyrosine phosphorylation [17] was used to develop a 96-well assay for stimulation and inhibition of ErbB4 tyrosine phosphorylation. Briefly, ErbB4 tyrosine phosphorylation was assayed using a “sandwich” enzyme-linked immunosorbent assay (ELISA) [18] kit (R&D Systems) that features an anti-ErbB4 capture antibody and an anti-phosphotyrosine detection antibody.

Lysates generated by a previously established protocol for stimulating ErbB4 tyrosine phosphorylation in CEM/ErbB4 cells [7] were used to develop and evaluate the ELISA conditions. Briefly, the CEM/ErbB4 cells were starved for 24 hours in serum- and factor-free base medium (RPMI) before being stimulated for seven minutes on ice with NRG2β (full agonist positive control), NRG2β/Q43L (partial agonist positive control), or NRG2β diluent (mock stimulation negative control). The cells were then lysed, and the lysates were immediately assayed for ErbB4 tyrosine phosphorylation or stored at -80°C until assayed.

**Fig 1:**
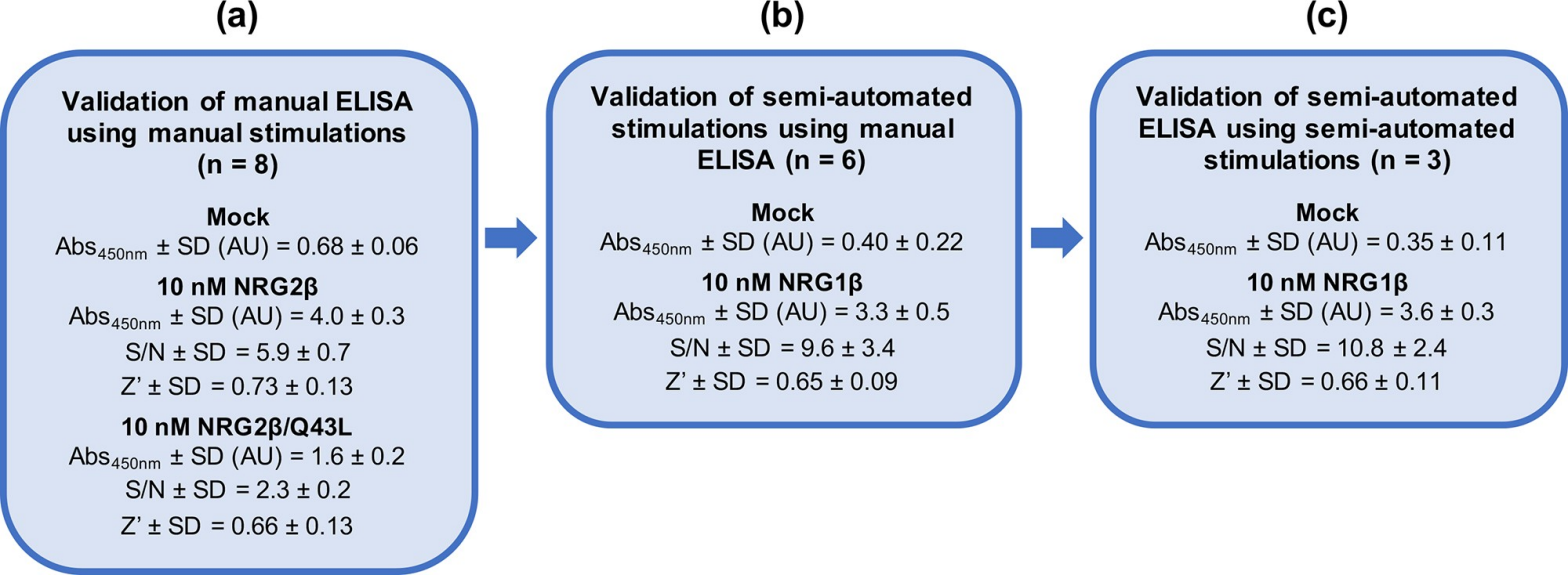
Validation of a semi-automated process for the stimulation and detection of ErbB4 tyrosine phosphorylation. (a) CEM/ErbB4 cells were manually stimulated with 10 nM NRG2β (full agonist positive control), 10 nM NRG2β/Q43L (partial agonist positive control), and mock (negative control). A single batch of lysate was assayed for ErbB4 tyrosine phosphorylation in eight independent trials using a manual sandwich ELISA. (b) CEM/ErbB4 cells were stimulated in six independent trials using a semi-automated protocol with 10 nM NRG1β (positive control) and mock (negative control). Each batch of lysate was assayed for ErbB4 tyrosine phosphorylation using a manual sandwich ELISA. (c) CEM/ErbB4 cells were stimulated using a semi-automated protocol with 10 nM NRG1β (positive control) and mock (negative control). A single batch of lysate was assayed for ErbB4 tyrosine phosphorylation in three independent trials using a semi-automated sandwich ELISA.

To evaluate the ELISA assay conditions, ErbB4 tyrosine phosphorylation stimulated by 10 nM NRG2β, 10 nM NRG2β/Q43L, or diluent (mock stimulation) was analyzed in eight independent ELISAs using a single batch of each of the three different lysates (Fig 1A). Stimulation by 10 nM NRG2β produces an average absorbance at 450 nm of 4.0 ± 0.3 AU, stimulation by 10 nM NRG2β/Q43L produces an average absorbance at 450 nm of 1.6 ± 0.2 AU, and mock stimulation produces an average absorbance at 450 nm of 0.68 ± 0.06 AU.

Using the effects of mock stimulation as the background levels of ErbB4 tyrosine phosphorylation, we determined that the average signal:noise ratio for stimulation by 10 nM NRG2β is 5.9 ± 0.7 and the average signal:noise ratio for stimulation by 10 nM NRG2β/Q43L is 2.3 ± 0.2 (Fig 1A).

The ability of the phospho-ErbB4 ELISA to reproducibly measure ligand-induced ErbB4 tyrosine phosphorylation was formally evaluated by calculating the Z’-factor (Z’) values for stimulation by 10 nM NRG2β or 10 nM NRG2β/Q43L. The Z’-factor is commonly used to evaluate the robustness of a high-throughput assay and is calculated using the means (μ) and standard deviations (σ) of the treatment samples (p) and untreated or negative (n) controls [19].

$$Z'‐\mathrm{factor}=1-\frac{3\left(\sigma_{p}+\sigma_{n}\right)}{\left|\mu_{p}-\mu_{n}\right|}$$ (1)

The maximum theoretical Z’-factor value is 1, whereas a Z’-factor value between 0.5 and 1.0 is indicative of a robust assay. A Z’-factor between 0 and 0.5 is indicative of a marginal assay [19]. Using data from eight independent ELISAs with three samples per treatment condition, stimulation with 10 nM of the ErbB4 full agonist (NRG2β) yields an average Z’-factor of 0.73 and stimulation with 10 nM of the ErbB4 partial agonist (NRG2β/Q43L) yields an average Z’-factor of 0.66 (Fig 1A). Thus, stimulation with either 10 nM NRG2β or 10 nM NRG2β/Q43L yields robust, reproducible levels of ErbB4 tyrosine phosphorylation as measured by the sandwich ELISA. Thus, this ELISA is appropriate for measuring stimulation and inhibition of ErbB4 tyrosine phosphorylation.

#### 2.1.2. Validation of a semi-automated process for the stimulation of ErbB4 tyrosine phosphorylation in a 96-well format

In order to develop an assay with the throughput needed to screen compound libraries, it was necessary to convert our standard batch method for agonist stimulation of ErbB4 tyrosine phosphorylation [7] to a semi-automated 96-well methodology. A Beckman Coulter Biomek 4000 automated liquid handling system was used to execute a semi-automated stimulation protocol that is based on our established batch protocol [7]. (The liquid-handling script for this procedure using the Biomek 4000 is available upon request.) We evaluated the robustness of this semi-automated stimulation protocol by using our validated phospho-ErbB4 ELISA assay to measure ErbB4 phosphorylation. CEM/ErbB4 cells were stimulated with 10 nM of the ErbB4 full agonist NRG1β or the NRG1β diluent (0.1% BSA in PBS) and ErbB4 tyrosine phosphorylation was assayed in wells that each contain a lysate generated from 3x10^5^ cells (Fig 1B). Based on six independent trials that each utilized three wells for each experimental condition, stimulation with NRG1β (10 nM) produces an average absorbance at 450 nm of 3.3 ± 0.5 AU and mock stimulation produces an average absorbance at 450 nm of 0.40 ± 0.22 AU. Thus, the average signal:noise ratio for stimulation with NRG1β (10 nM) is 9.6 ± 3.4. It should be noted that the signal:noise ratio for semi-automated stimulation with 10 nM NRG1β is almost twice the signal:noise ratio for manual stimulation with 10 nM NRG2β (Fig 1B). This appears to be largely due to the decreased amount of (background) ErbB4 tyrosine phosphorylation stimulated by the diluent negative control. Furthermore, the six trials of semi-automated stimulation with 10 nM NRG1β yields an average ErbB4 tyrosine phosphorylation Z’-factor (Z’) score in excess of 0.6 (Fig 1B), which indicates that semi-automated stimulation with 10 nM NRG1β yields robust and reproducible ErbB4 tyrosine phosphorylation that is suitable for deployment in a high-throughput workflow.

#### 2.1.3. Validation of a semi-automated phospho-ErbB4 sandwich ELISA in 96-well format for the detection of ErbB4 tyrosine phosphorylation

In an attempt to make additional improvements to throughput and reproducibility, we developed and evaluated a semi-automated sandwich ELISA protocol in a 96-well format. Once again, the Biomek 4000 was used to adapt the existing phospho-ErbB4 ELISA protocol to a semi-automated workflow. (The liquid-handling script for this procedure using the Biomek 4000 is available upon request.) The ErbB4 full agonist NRG1β (10 nM) was used as the positive control for stimulation of ErbB4 tyrosine phosphorylation and the NRG1β diluent (0.1% BSA in PBS) was used as the negative control. For comparison purposes, non-automated and semi-automated ELISAs were performed on wells that each contain a lysate prepared from 3x10^5^ cells. In three independent semi-automated ELISAs performed using a single batch of lysate (Fig 1C) and three wells for each experimental condition, stimulation with NRG1β (10 nM) yields an average absorbance at 450 nm of 3.6 ± 0.3 AU and stimulation with NRG1β diluent (mock) yields an average absorbance at 450 nm of 0.35 ± 0.11 AU. Based on these results, the average signal:noise ratio for stimulation with 10 nM NRG1β is 10.8 ± 2.4. Thus, the fully semi-automated process exhibits the highest S/N and the lowest noise levels of all three techniques that were used (Fig 1). Furthermore, assaying stimulation of ErbB4 phosphorylation (by 10 nM NRG1β) using the semi-automated ELISA yields an average Z’-factor (Z’) score of 0.66. Therefore, the semi-automated processes for stimulation and detection of ErbB4 tyrosine phosphorylation are suitable for use in a high throughput screening process. Given the cost of the ELISA assay reagents and other consumables, and based on three replicates per sample and three independent trials, it costs $15 to test a single concentration of a single compound for stimulation of ErbB4 tyrosine phosphorylation. Because of this expense, the ELISA-based screen for compounds that stimulate ErbB4 tyrosine phosphorylation can be performed on only a relatively small number of compounds. Thus, this screen must lie downstream of less expensive strategies for screening relatively large numbers of compounds.

### 2.2. ErbB4-dependent cellular proliferation can be stimulated and detected via semi-automated 96-well assays

Because our ultimate goal is the identification of compounds that inhibit ErbB4-dependent cell proliferation, we sought to develop a semi-automated 96-well assay for ErbB4-dependent cell proliferation that could be deployed to identify inhibitors.

Mouse BaF3 pro-B-lymphocyte cells do not endogenously express EGFR, ErbB2, or ErbB4 and their survival and proliferation requires the exogenous growth factor interleukin-3 (IL3). However, a BaF3 cell line that ectopically expresses human EGFR and ErbB4 exhibits IL3-independent proliferation upon stimulation with the ErbB4 full agonist NRG1β. This proliferation is dependent upon exogenous ErbB4 expression [8]. Therefore, the BaF3/EGFR+ErbB4 cell line [8] was used to assay ErbB4-dependent, IL3-independent cell proliferation (henceforth referred to as ErbB4-dependent proliferation).

We used an established manual 24-well assay [8] as the basis for developing a semi-automated 96-well assay for stimulation and detection of ErbB4-dependent cellular proliferation. (The liquid-handling script for this procedure using the Biomek 4000 is available upon request.) The ErbB4 full agonist NRG1β was used as the positive control. Briefly, BaF3/EGFR+ErbB4 cells were grown to saturation (2.5x10^6^ cells/mL) and aseptically seeded at 1x10^4^ cells/well (100 μL @ 1x10^5^ cells/mL) into a sterile 96-well microplate in the absence of IL3. The cells were then treated with increasing concentrations of NRG1β in triplicate. The plate was incubated at 37°C and 5% CO_2_ for 120 hours before cell proliferation was analyzed using an MTT assay [20]. Briefly, cells were treated by directly adding 10 μL of MTT Reagent (3-(4,5-dimethylthiazol-2-yl)-2,5-diphenyltetrazolium bromide; ATCC^®^ 30-1010K™) to the culture medium in each well and incubated in the dark for two hours at 37°C and 5% CO_2_. Then, 100 μL of Detergent Reagent (ATCC^®^ 30-1010K™) was added to each well and the plate was incubated in the dark overnight at room temperature. MTT conversion was then determined by measuring the absorbance of each well at 570 nm. The dose response data were then analyzed using GraphPad Prism to determine the EC_50_ and E_max_ for NRG1β. In this assay, MTT conversion is a function of viable cell number, not a function of cell metabolism (S1 Fig).

**Fig 2:**
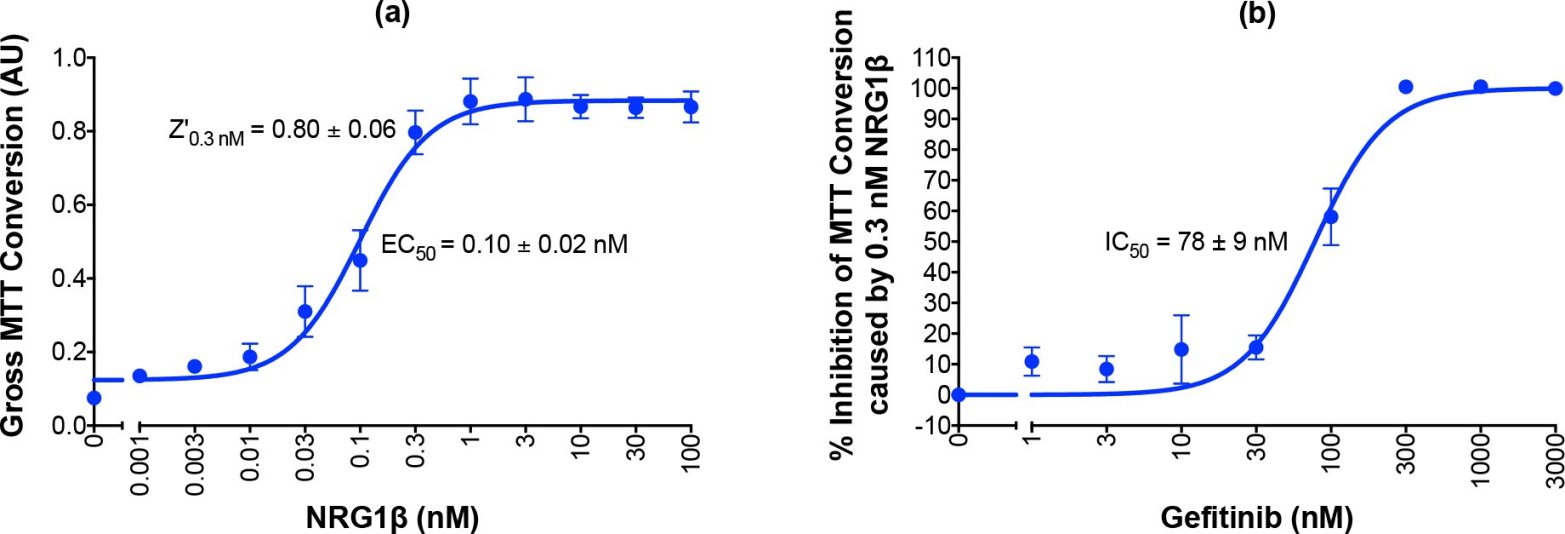
Validation of semi-automated processes for the stimulation of ErbB4-dependent cellular proliferation and detection of the inhibition of agonist-induced ErbB4-dependent cellular proliferation. (a) BaF3/EGFR+ErbB4 cells were stimulated with increasing concentrations of NRG1β in four independent trials using a semi-automated protocol. A semi-automated MTT assay was used to analyze cell proliferation 120 hours post-stimulation. Curves were fit to the data using GraphPad Prism to determine the EC_50_ of NRG1β. The Z-factor for ErbB4-dependent cell proliferation stimulated by 0.3 nM NRG1β was calculated using the means and standard deviations of the positive (0.3 nM NRG1β) and negative (NRG1β-diluent) controls. (b) BaF3/EGFR+ErbB4 cells were treated with increasing concentrations of gefitinib in the presence of 0.3 nM NRG1β in four independent trials using a semi-automated protocol. A semi-automated MTT assay was used to analyze cell proliferation 120 hours post-stimulation. Curves were fit to the data using GraphPad Prism to determine the IC_50_ of gefitinib against NRG1β.

In four independent trials (Fig 2A) with three wells per treatment condition, NRG1β exhibits an average EC_50_ of 0.10 ± 0.02 nM. Furthermore, stimulation with 0.3 nM NRG1β yielded an average ErbB4-dependent cell proliferation Z-factor score of 0.80 ± 0.06. This Z-factor calculation verifies that NRG1β-induced ErbB4-dependent cellular proliferation can be reproducibly measured when BaF3/EGFR+ErbB4 cells are stimulated with 0.3 nM NRG1β. Thus, this semi-automated assay is suitable for identifying compounds that stimulate ErbB4-dependent cell proliferation. Given the cost of consumables, and based on three replicates per sample and three independent trials, it costs $2 to test a single concentration of a single compound for stimulation of ErbB4-dependent cell proliferation.

### 2.3. The inhibition of agonist-induced ErbB4-dependent cellular proliferation can be detected via semi-automated 96-well assays

In multiple model systems (including BaF3 cells), ErbB4-dependent cellular proliferation is dependent upon ErbB4 heterodimerization with EGFR or ErbB2 and ErbB4 phosphorylation by EGFR or ErbB2 [7–13, 21]. To be explicit, experiments using mutants defective for kinase activity and kinase inhibitors demonstrate that EGFR or ERBB2 kinase activity, but not ErbB4 kinase activity, is required for EGFR-ErbB4 heterodimers or ErbB2-ErbB4 heterodimers to stimulate cell proliferation [7, 21]. Consequently, an EGFR kinase inhibitor will inhibit stimulation of cell proliferation by an EGFR-ERBB4 heterodimer only through inhibition of EGFR kinase activity, not through inhibition of ERBB4 kinase activity [7].

Therefore, we explored whether our semi-automated assay for ErbB4-dependent proliferation (Fig 2A) could be adapted to detect inhibition of ErbB4-dependent proliferation of the BaF3/EGFR+ErbB4 cell line by the EGFR tyrosine kinase inhibitor gefitinib (Fig 2B). (The liquid-handling script for this procedure using the Biomek 4000 is available upon request.) BaF3/EGFR+ErbB4 cells were treated with increasing concentrations of gefitinib in the presence of 0.3 nM NRG1β, a concentration of NRG1β that provides robust and highly reproducible levels of ErbB4-dependent cellular proliferation (Fig 2A). Four independent trials demonstrate that gefitinib completely inhibits stimulation of proliferation by 0.3 nM NRG1β and yields an IC_50_ for gefitinib of 78 ± 9 nM; a value that is comparable to previously reported IC_50_ values for gefitinib [22, 23]. Furthermore, these trials demonstrated that gefitinib markedly and reproducibly inhibits the stimulation of proliferation of BaF3/EGFR+ErbB4 cells by NRG1β; the average Z-factor resulting from treatment with 100 nM gefitinib is 0.26 ± 0.45, whereas the average Z-factor resulting from treatment with 300 nM gefitinib is 0.74 ± 0.21. These Z-factor calculations indicate that 100 nM and 300 nM gefitinib both inhibit the proliferation of BaF3/EGFR+ErbB4 cells, but that 300 nM gefitinib is more suitable for reproducibly measuring the inhibition of BaF3/EGFR+ErbB4 cell proliferation. Thus, this semi-automated proliferation assay can be used for the high-throughput identification and characterization of compounds that inhibit agonist-induced ErbB4-dependent cellular proliferation and 300 nM gefitinib can serve as a positive control for inhibition of agonist-induced ErbB4-dependent cellular proliferation.

### 2.4. Screening process for the identification of small molecule compounds that function as inhibitors of agonist-induced ErbB4-dependent cellular proliferation

#### 2.4.1. Overview

**Fig 3:**
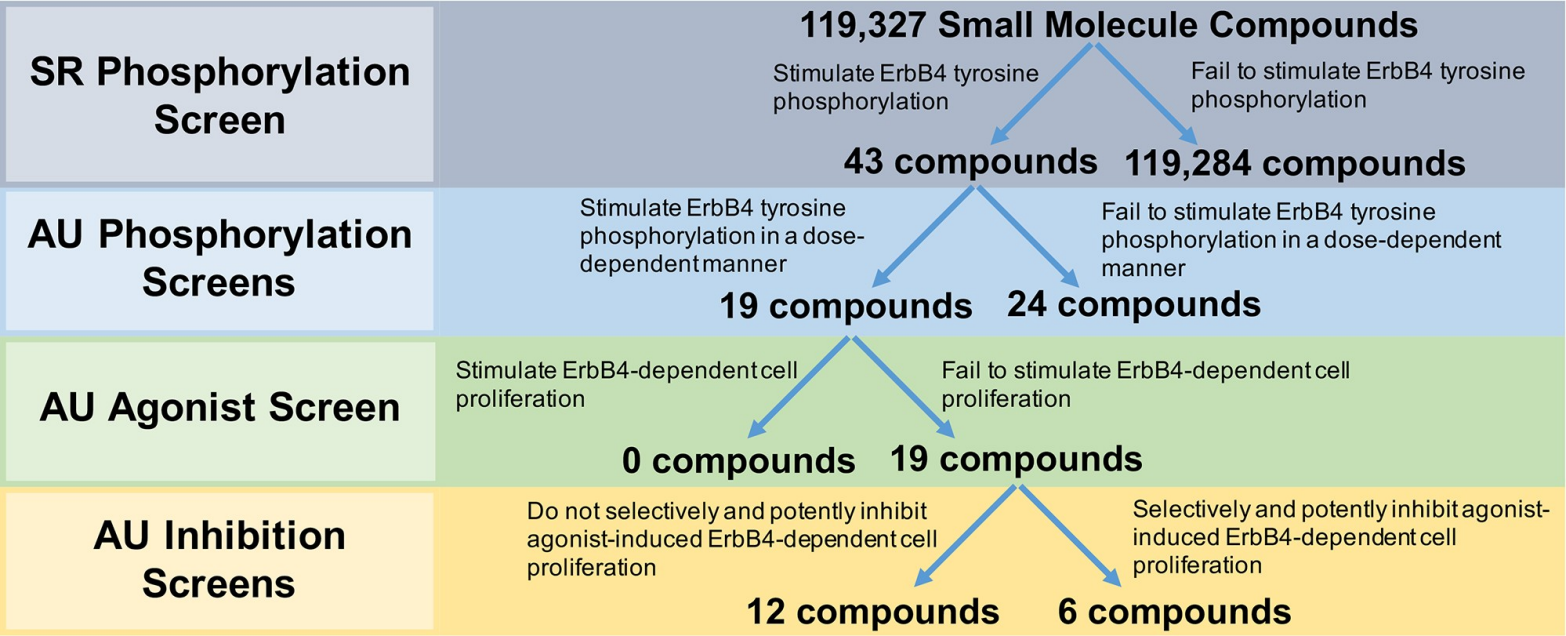
Deployment strategy of screening methodologies for the identification of partial agonists at the ErbB4 receptor tyrosine kinase that function as ErbB4 antagonists. Southern Research (SR) Phosphorylation Screen: SR screened a library of small molecule compounds (~100k) for stimulation of ErbB4 tyrosine phosphorylation using an ultra-high throughput assay developed by DiscoverX. Auburn University (AU) Phosphorylation Screens: The 43 most promising compounds identified from the high-throughput SRI screen were then tested for concentration-dependent stimulation of ErbB4 tyrosine phosphorylation. AU Agonist Screen: The compounds that exhibited dose-dependent stimulation of ErbB4 tyrosine phosphorylation were tested to determine whether they stimulated ErbB4-dependent cellular proliferation. AU Inhibition Screens: The compounds that failed to stimulate ErbB4-dependent cellular proliferation were tested to determine whether they inhibited agonist-induced ErbB4-dependent cellular proliferation. Selectivity of these inhibitors was determined by comparing inhibition of ErbB4-dependent cellular proliferation against inhibition of Interleukin 3-dependent (IL3-dependent) cellular proliferation.

Our strategy for deploying our screens to identify compounds that may function as inhibitors of ErbB4-dependent proliferation is described in Fig 3. As noted elsewhere, our strategy is intended to identify ErbB4 partial agonists; such molecules stimulate ErbB4 tyrosine phosphorylation, yet fail to stimulate ErbB4-dependent cell proliferation and inhibit stimulation of ErbB4-dependent cell proliferation by an ErbB4 agonist.

To identify ErbB4 agonists, a high throughput screen (HTS) of a structurally diverse set of 119,327 small molecule compounds selected from the compound collection maintained at Southern Research (Birmingham, Alabama) was performed using the DiscoverX PathHunter® U2OS ErbB4 Functional Assay to measure stimulation of ErbB4 tyrosine phosphorylation [24]. This assay utilizes a cell line (U2OS) that is engineered to express a recombinant ErbB4 that has been tagged with a ProLink™ (PK) epitope at the intracellular carboxyl terminus of the receptor. The cell line also expresses a recombinant SH2 domain that is selective for binding to phosphorylated ErbB4 and has been fused to an Enzyme Acceptor (EA) epitope. Upon phosphorylation of the recombinant ErbB4-ProLink molecule, it binds the SH2-EA and the proximity of the two recombinant proteins creates an active β-galactosidase (β-gal) enzyme. β-gal activity is quantified using a chemiluminescent substrate. This β-gal “complementation” assay ensures specificity for ErbB4 tyrosine phosphorylation. However, this strategy will detect ligand-independent (basal) ErbB4 tyrosine phosphorylation, necessitating the use of vehicle-treated cells as a negative (background) control and rigorous confirmatory assays (for example, the phospho-ErbB4 ELISA assay) to rule out false positives.

Using this platform, stimulation with the ErbB4 full agonist NRG1β (100nM) resulted in a two-fold increase in signal compared to negative control cells treated with vehicle. A total of 32 positive and 32 negative control wells were included in each of the 400 plates run in the HTS campaign and the signals resulted in an average Z’-factor of 0.33. The DiscoverX PathHunter® U2OS ErbB4 Functional Assay is considerably cheaper on a per-compounds basis than the ELISA assay for ErbB4 tyrosine phosphorylation described in Section 2.1.3. Thus, despite the fact that the DiscoverX PathHunter® U2OS ErbB4 Functional Assay yields lower Z’-factor values than does the ELISA assay for ErbB4 tyrosine phosphorylation, the DiscoverX PathHunter® U2OS ErbB4 Functional Assay is a much better choice for performing the screen of the 119,327 molecules in the selected library.

Using two-fold stimulation as the threshold for compound activity, the HTS identified 132 compounds that stimulated ErbB4 tyrosine phosphorylation. Of these, 43 compounds were confirmed by exhibiting concentration-dependent activity when retested over a concentration range of 0.1 to 60 μg/ml. These 43 compounds were then carried forward for further evaluation using the strategies described previously in this work.

### 2.4.2. Nineteen candidates stimulated ErbB4 tyrosine phosphorylation in a dose-dependent manner

**Fig 4:**
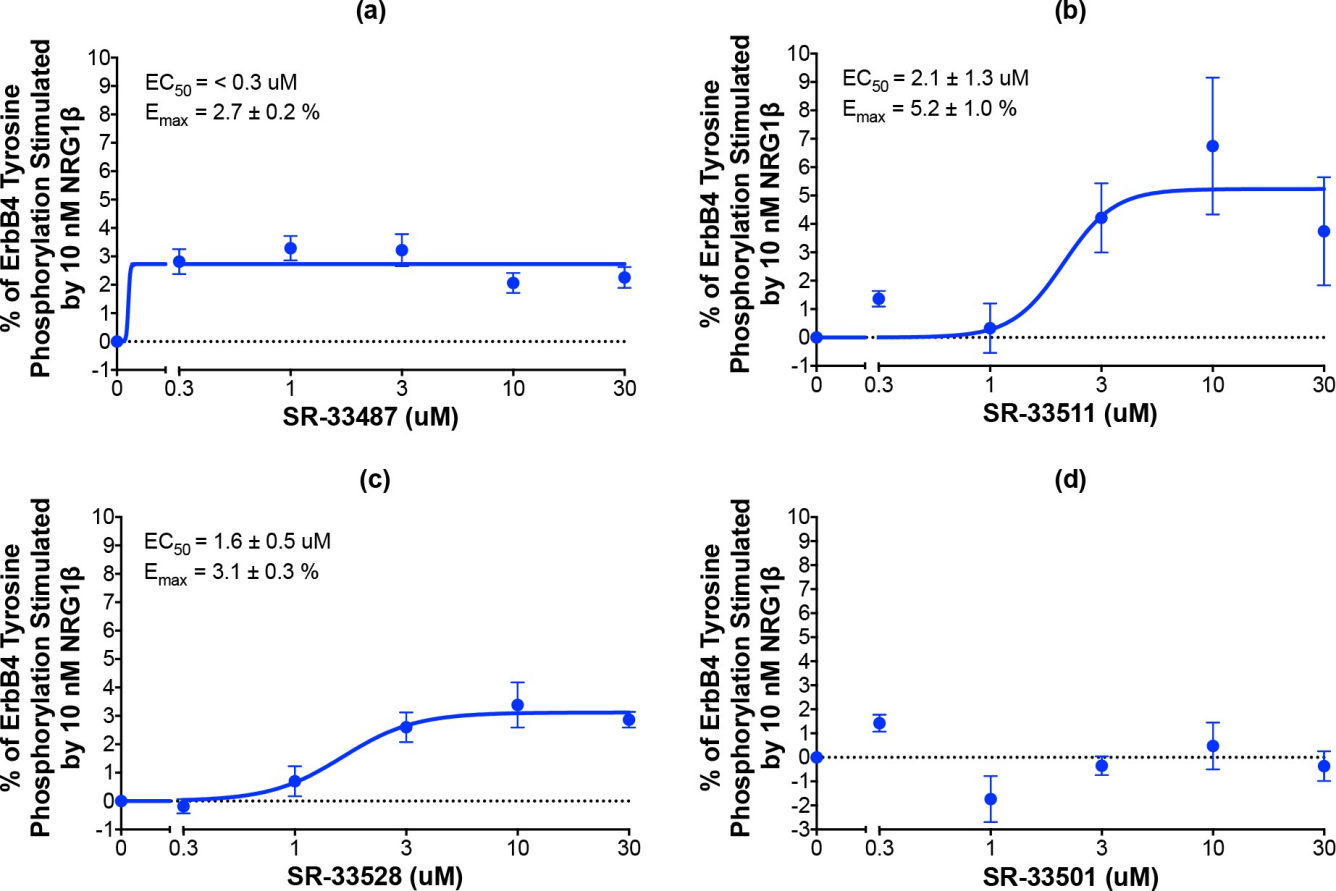
Representative candidates that stimulate and fail to stimulate ErbB4 tyrosine phosphorylation in a dose-dependent manner. (a-d) In at least three independent trials and using semi-automated processes, CEM/ErbB4 cells were stimulated with increasing concentrations of the candidate ErbB4 ligands. ErbB4 tyrosine phosphorylation was assayed using the semi-automated sandwich ELISA. Curves were fit to the data using GraphPad Prism to determine the EC_50_ and E_max_ values for stimulation of ErbB4 tyrosine phosphorylation by each candidate. EC_50_ and E_max_ values are also shown in Table 1.

All 43 compounds that stimulate ErbB4 tyrosine phosphorylation were tested at a single concentration (10 μM) for stimulation of ErbB4 tyrosine phosphorylation using our semi-automated ELISA-based screening process (n = 3). Compounds that stimulated ErbB4 tyrosine phosphorylation in at least two of three independent trials were subsequently tested at a range of concentrations (0–30 μM) in at least three independent trials to determine whether the compounds stimulated ErbB4 tyrosine phosphorylation in a concentration-dependent manner. The data obtained from testing three of the compounds that stimulated ErbB4 tyrosine phosphorylation in a concentration-dependent manner are shown in Fig 4A–4C. The data obtained from testing one of the compounds that did not stimulate ErbB4 tyrosine phosphorylation in a concentration-dependent manner are shown in Fig 4D. Based on the results of these experiments, 19 compounds were judged to stimulate ErbB4 tyrosine phosphorylation and 24 were judged to be incapable of stimulating ErbB4 tyrosine phosphorylation (Fig 3). The apparent false positivity rate (56%) of the DiscoverX PathHunter® U2OS ErbB4 Functional Assay illustrates the shortcoming of using this assay as the only means for identifying candidate ErbB4 partial agonists and the need for rigorous confirmatory assays to rule out false positives.

The EC_50_ and E_max_ were calculated for each of the 19 compounds that stimulated ErbB4 tyrosine phosphorylation (Table 1). The most potent compounds exhibit an EC_50_ of less than 0.3 μM and the most efficacious compounds exhibit an Emax in excess of 5% of the amount of ErbB4 tyrosine phosphorylation stimulated by 10 nM NRG1β.

**Table 1:** Stimulation of ErbB4 tyrosine phosphorylation by candidates.

SR ID #	ErbB4 Tyrosine Phosphorylation	
	EC_50_ ± SE (μM)	E_max_ ± SE (%)^†^
SR-33486	< 0.3	5.9 ± 2.8
SR-33511	2.1 ± 1.3	5.2 ± 1.0
SR-33483	0.47 ± 0.38	4.7 ± 1.1
SR-33520	< 0.3	3.3 ± 1.0
SR-33528	1.6 ± 0.5	3.1 ± 0.3
SR-33485	< 0.3	3.0 ± 0.9
SR-33507	< 0.3	2.9 ± 0.4
SR-33491	0.68 ± 0.36	2.8 ± 0.4
SR-33519	< 0.3	2.7 ± 0.3
SR-33487	< 0.3	2.7 ± 0.2
SR-33513	< 0.3	2.4 ± 0.2
SR-33494	< 0.3	2.3 ± 0.4
SR-33493	< 0.3	2.1 ± 3.8
SR-33497	< 0.3	1.8 ± 0.5
SR-33492	4.1 ± 2.9	1.8 ± 0.2
SR-33509	0.38 ± 0.25	1.4 ± 0.3
SR-33498	1.5 ± 0.6	1.4 ± 0.4
SR-33502	< 0.3	1.4 ± 0.4
SR-33510	2.0 ± 0.8	1.2 ± 0.4

^†^Maximal ErbB4 tyrosine phosphorylation stimulated by candidates as a percentage of ErbB4 tyrosine phosphorylation stimulated by 10 nM NRG1β.

#### 2.4.3. Nineteen candidates inhibit agonist-induced ErbB4-dependent cellular proliferation

**Fig 5:**
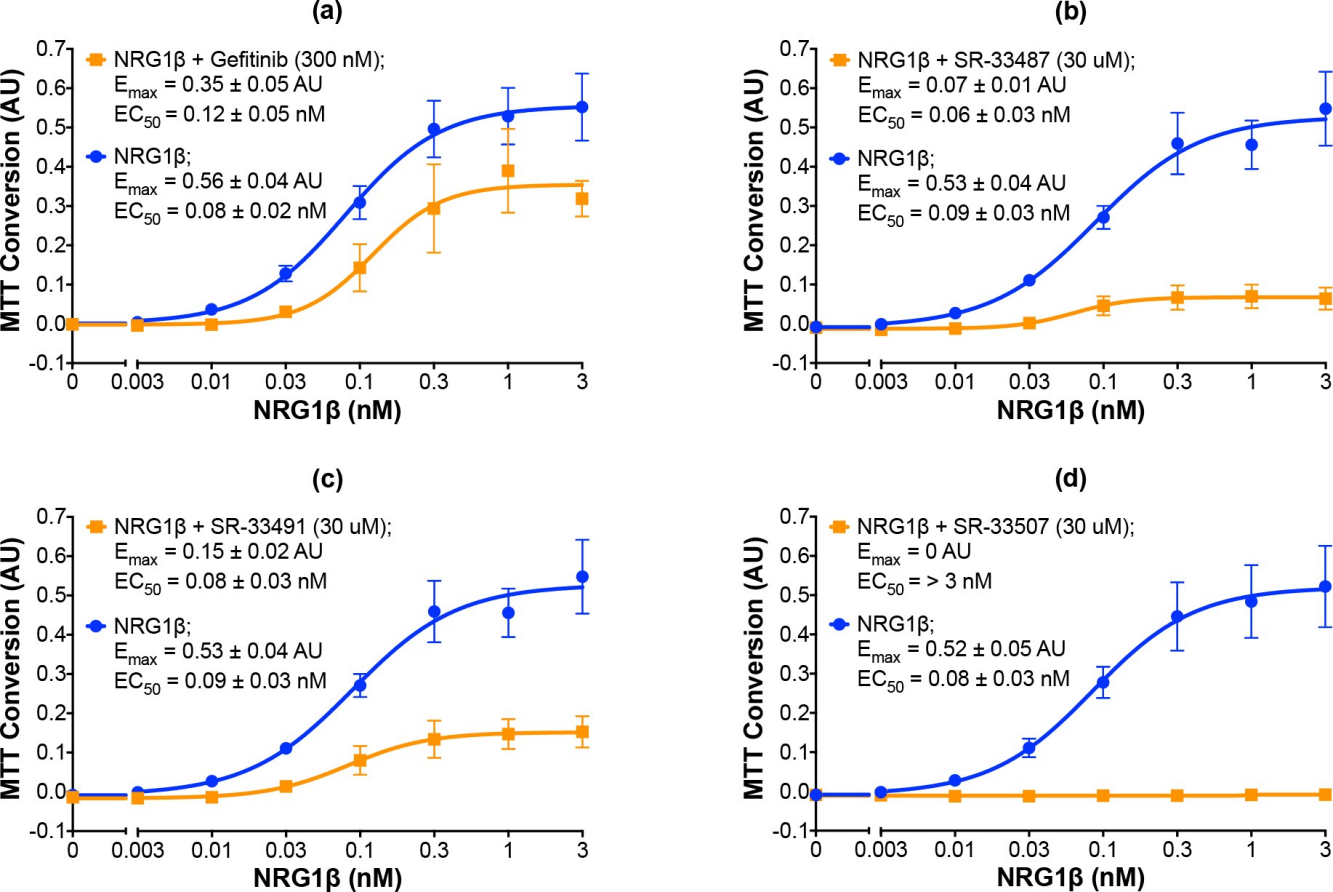
Representative candidates that inhibit agonist-induced ErbB4-dependent cellular proliferation. (a-d) In three independent trials and using semi-automated processes, BaF3/EGFR+ErbB4 cells were stimulated with increasing concentrations of NRG1β in the presence or absence of gefitinib at 300 nM or candidates at 30 uM. A semi-automated MTT assay was used to analyze cell proliferation at 120 hours post-stimulation. Curves were fit to the data using GraphPad Prism to determine the EC_50_ and E_max_ of NRG1β in the presence and absence of gefitinib or the candidates. EC_50_ and E_max_ values are also shown in Table 2.

The 19 compounds that stimulated ErbB4 tyrosine phosphorylation in a concentration-dependent manner were then tested at a single concentration (30 μM) in three independent trials to determine whether any of the compounds also stimulated ErbB4-dependent cellular proliferation. Using our semi-automated MTT assay, we determined that all 19 compounds fail to stimulate any ErbB4-dependent cellular proliferation (Fig 3). Consequently, each of these 19 compounds were then tested at a single concentration (30 μM) in combination with increasing concentrations of an ErbB4 full agonist (NRG1β) to determine whether these compounds are capable of inhibiting agonist-induced ErbB4-dependent cellular proliferation. Based on the data shown in Fig 2B, gefitinib (300 nM) was used as a positive control for inhibition of agonist-induced ErbB4-dependent cellular proliferation (Fig 5A). At 300 nM, gefitinib reduces the E_max_ for NRG1β from 0.56 ± 0.04 AU to 0.35 ± 0.05 AU. Gefitinib shifts the EC_50_ for NRG1β from 0.08 ± 0.02 nM to 0.12 ± 0.05 nM; this very minor decrease in agonist potency is consistent with the observation that gefitinib does not directly compete with NRG1β for binding to the EGFR-ErbB4 heterodimer.

Three independent trials reveal that the candidate ErbB4 inhibitors exhibit a wide distribution of inhibitory effects on stimulation of ErbB4-dependent cellular proliferation by NRG1β (Table 2). Some candidates exhibit modest inhibitory efficacy, such as SR-33513 (Table 2). In contrast, other compounds exhibit greater efficacy, such as SR-33487, which inhibits the NRG1β E_max_ by 87% (Fig 5B and Table 2) and SR-33491, which inhibits the NRG1β E_max_ by 72% (Fig 5C and Table 2). Indeed, 30 μM of some compounds, such as SR-33507, completely inhibits cellular proliferation stimulated by NRG1β (Fig 5D and Table 2). Despite the fact that the compounds exhibit varying effects on NRG1β efficacy, none altered the potency of NRG1β in a noteworthy manner. This suggests that none of the compounds compete with NRG1β for binding to ErbB4. Nonetheless, all 19 of these compounds stimulate ErbB4 tyrosine phosphorylation in a concentration-dependent manner, fail to stimulate ErbB4-dependent cellular proliferation, and inhibit agonist-induced ErbB4-dependent cellular proliferation to some degree.

**Table 2:** Effect of candidates on stimulation of cell proliferation by NRG1β.

Candidate (30 μM)	NRG1β E_max_ ± SE (AU)	NRG1β EC_50_ ± SE (nM)
None	0.54 ± 0.02	0.08 ± 0.01
Gefitinib (300 nM)	0.35 ± 0.05	0.12 ± 0.05
SR-33483	0	> 3
SR-33486	0	> 3
SR-33493	0	> 3
SR-33494	0	> 3
SR-33498	0	> 3
SR-33507	0	> 3
SR-33511	0	> 3
SR-33528	0	> 3
SR-33485	0.01 ± 0.02	0.52 ± 0.71
SR-33510	0.03 ± 0.01	0.11 ± 0.08
SR-33492	0.03 ± 0.01	0.15 ± 0.11
SR-33487	0.07 ± 0.01	0.06 ± 0.03
SR-33497	0.09 ± 0.02	0.05 ± 0.03
SR-33509	0.11 ± 0.02	0.09 ± 0.06
SR-33491	0.15 ± 0.02	0.08 ± 0.03
SR-33519	0.18 ± 0.03	0.08 ± 0.04
SR-33520	0.26 ± 0.04	0.09 ± 0.05
SR-33502	0.34 ± 0.04	0.06 ± 0.02
SR-33513	0.41 ± 0.05	0.09 ± 0.04

#### 2.4.4. Six candidates appear to selectively and potently inhibit ErbB4 coupling to cell proliferation

**Fig 6:**
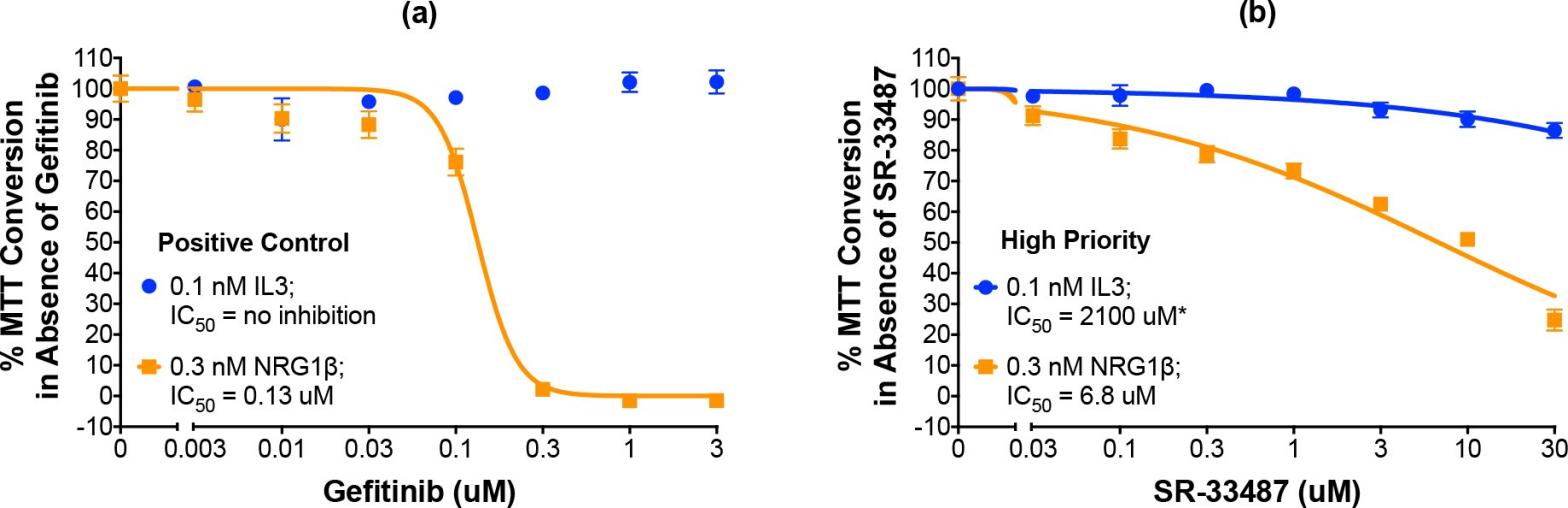
A high-priority small molecule compound selectively and potently inhibits ErbB4-dependent cellular proliferation. (a-b) In three independent trials and using a modified version of our semi-automated processes, BaF3/EGFR+ErbB4 cells were treated with increasing concentrations of a candidate inhibitor or gefitinib (positive control inhibitor of NRG1β-induced proliferation) in the presence of 0.1 nM IL3 or 0.3 nM NRG1β. A semi-automated MTT assay was used to analyze cellular proliferation 120 hours post-stimulation. Curves were fit to the data using GraphPad Prism to determine the IC_50_ value for each inhibitor against 0.1 nM IL3 and 0.3 nM NRG1β. IC_50_ values are also shown in Table 3. *Extrapolated value.

As noted earlier, the BaF3/EGFR+ErbB4 cells are IL3 dependent and this dependency can be rescued by stimulation of ErbB4 coupling to cell proliferation [8]. Therefore, the specificity of each of the 19 candidate ErbB4 inhibitors was tested by assaying inhibition of ErbB4-dependent and IL3-dependent cellular proliferation in parallel. Using a slightly modified version of our existing automated protocol, we tested the effects of increasing concentrations of each compound on the effects of 0.3 nM NRG1β or 0.1 nM IL3 on proliferation of the BaF3/EGFR+ErbB4 cell line. Gefitinib completely and potently (IC_50_ of 0.13 μM) inhibits the effect of stimulation with 0.3 nM NRG1β yet fails to inhibit the effect of stimulation with 0.1 nM IL3. Therefore, gefitinib serves as the positive control for selective inhibition of ErbB4-dependent cellular proliferation (Fig 6A, Table 3).

**Table 3:** Effect of increasing concentrations of candidate inhibitors on stimulation of cell proliferation by 0.3 nM NRG1β or 0.1 nM IL3.

Candidate	IC_50_ (μM) against 0.3 nM NRG1β	IC_50_ (μM) against 0.1 nM IL3	Priority
Gefitinib	0.13	NA	Control
SR-33528	< 0.03	< 0.03	Special Case
SR-33486	0.11	0.45	Medium
SR-33507	0.39	0.72	Low
SR-33498	2.3	4.8	Low
SR-33493	5.6	15	Low
SR-33483	6.0	28	Medium
SR-33511	6.8	13	Low
SR-33487	6.8	2100*	High
SR-33494	7.0	32*	Medium
SR-33510	11	29	Low
SR-33492	12	81*	Medium
SR-33485	13	38*	Low
SR-33491	29	NA	Medium
SR-33509	32*	NA	Low
SR-33497	47*	390*	Low
SR-33520	830*	NA	No
SR-33502	NA	NA	No
SR-33513	NA	NA	No
SR-33519	NA	NA	No

*Extrapolated value. NA = not active.

Following analyses of the data from three independent trials, the compounds were categorized based on the relative ability to selectively and potently inhibit ErbB4-dependent cellular proliferation. To summarize results which will be presented in the remainder of this section, six of the compounds selectively and potently inhibit agonist-induced ErbB4-dependent cellular proliferation, twelve of the compounds do not selectively and potently inhibit agonist-induced ErbB4-dependent cellular proliferation, and one compound is considered a special case.

**Fig 7:**
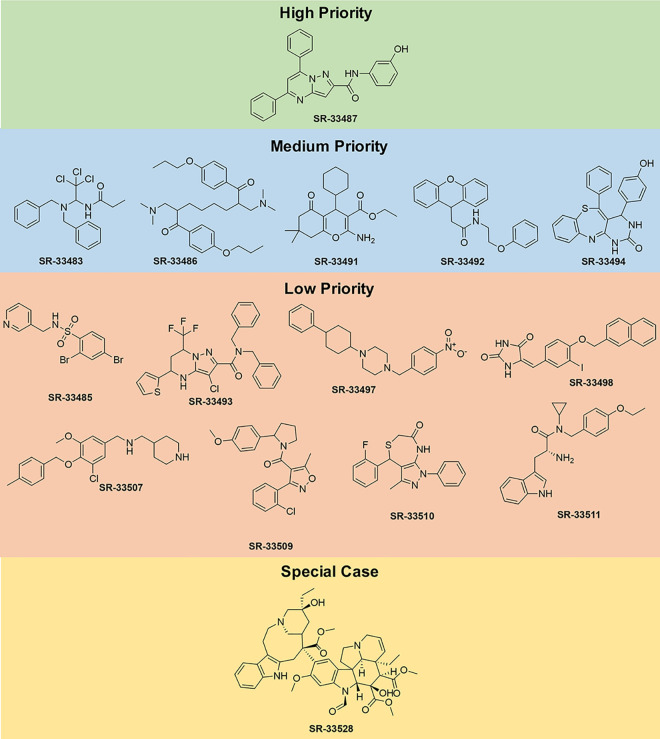
Chemical structures of high priority, medium priority, low priority, and special case compounds.

One compound is judged to be a high priority candidate based on an IC_50_ against NRG1β-induced ErbB4-dependent cell proliferation of less than 10 μM and a selectivity for ErbB4 over IL3 (the IC_50_ value against IL3 divided by the IC_50_ value against NRG1β—IC_50,IL3_:IC_50,NRG1β_) of greater than 10. The high-priority candidate, SR-33487, inhibits the stimulation of cell proliferation by 0.3 nM NRG1β with an IC_50_ of 6.8 μM and is predicted to inhibit IL3-dependent cellular proliferation with an IC_50_ of 2100 μM (Figs 6B and 7 and Table 3). Thus, SR-33487 is a much more potent inhibitor of ErbB4-dependent cell proliferation than of IL3-dependent cell proliferation.

**Fig 8:**
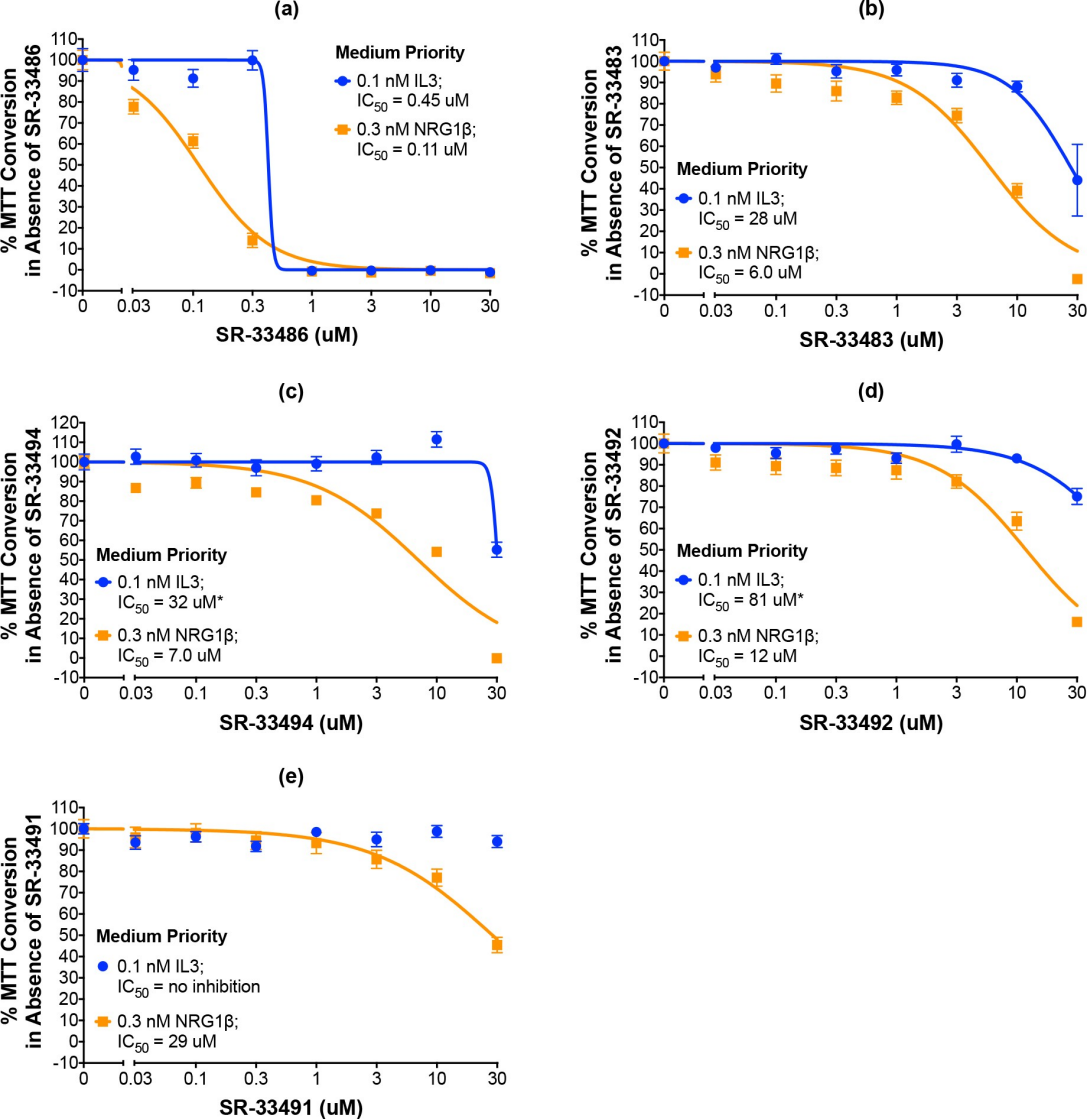
Five compounds are potent and selective inhibitors of ErbB4-dependent cellular proliferation. (a-e) In three independent trials and using a modified version of our semi-automated processes, BaF3/EGFR+ErbB4 cells were treated with increasing concentrations of each candidate inhibitor in the presence of 0.1 nM IL3 or 0.3 nM NRG1β. A semi-automated MTT assay was used to analyze cellular proliferation 120 hours post-stimulation. Curves were fit to the data using GraphPad Prism to determine the IC_50_ value for each candidate against 0.1 nM IL3 and 0.3 nM NRG1β. IC_50_ values are also shown in Table 3.

The remaining five compounds that selectively and potently inhibit ErbB4-dependent cell proliferation are considered medium priority candidates (Figs 7 and 8A–8E). Medium priority candidates exhibit an IC_50_ of less than 30 μM against NRG1β-induced ErbB4-dependent cell proliferation AND a selectivity for ErbB4 over IL3 of at least 3. For example, SR-33486 inhibits the stimulation of cell proliferation by 0.3 nM NRG1β with an IC_50_ of 0.11 uM and inhibits IL3-dependent cellular proliferation with an IC_50_ of 0.45 μM (Fig 8A and Table 3). Thus, even though SR-33486 is a more potent inhibitor of ErbB4-dependent cell proliferation than is the high priority candidate (SR-33487), SR-33486 is much less selective for ErbB4 than is SR-33487.

Eight compounds are judged to be low priority candidates (Fig 7, S2 and S3 Figs). Low priority candidates exhibit an IC_50_ of less than 30 μM against NRG1β-induced ErbB4-dependent cell proliferation OR a selectivity for ErbB4 over IL3 of at least 3. For example, SR-33507 inhibits the stimulation of cell proliferation by 0.3 nM NRG1β with an IC_50_ of 0.39 μM and inhibits IL3-dependent cellular proliferation with an IC_50_ of 0.72 μM (S2 Fig and Table 3). Also, SR-33509 inhibits the stimulation of cell proliferation by 0.3 nM NRG1β with an IC_50_ of 32 μM and fails to inhibit IL3-dependent cellular proliferation (S3 Fig and Table 3).

Our assays identified four compounds that do not selectively nor potently inhibit agonist-induced ErbB4-dependent cell proliferation and therefore possess no priority for further development (Fig 7 and S4 Fig and Table 3). In other words, these molecules exhibit an IC_50_ of GREATER than 30 μM against NRG1β-induced ErbB4-dependent cell proliferation AND a selectivity for ErbB4 over IL3 of LESS than 3.

#### 2.4.5. “Special case” molecule SR-33528 is not a selective inhibitor of ErbB4

**Fig 9:**
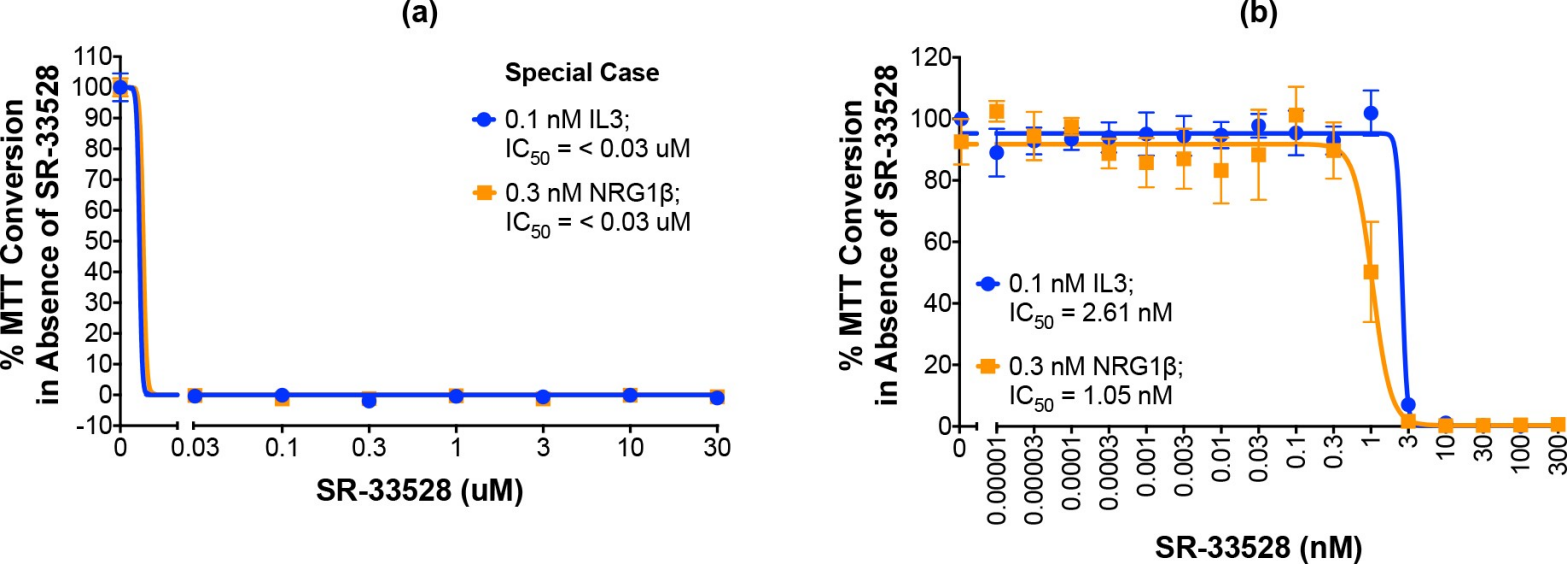
“Special case” molecule SR-33528 is not a selective ErbB4 inhibitor. (a-b) In three independent trials and using a modified version of our semi-automated processes, BaF3/EGFR+ErbB4 cells were treated with increasing concentrations of each candidate inhibitor in the presence of 0.1 nM IL3 or 0.3 nM NRG1β. A semi-automated MTT assay was used to analyze cellular proliferation 120 hours post-stimulation. Curves were fit to the data using GraphPad Prism to determine the IC_50_ value for each candidate against 0.1 nM IL3 and 0.3 nM NRG1β.

One molecule, SR-33528, is considered a special case as our initial analyses (Fig 9A; Table 3) indicate that it potently inhibits stimulation of cell proliferation by 0.3 nM NRG1β (IC_50_ of less than 30 nM) and by 0.1 nM IL3 (IC_50_ of less than 30 nM). Since this potent inhibition of ErbB4-dependent and IL3-dependent proliferation made it impossible to judge the selectivity of this candidate for inhibition of ErbB4-dependent cell proliferation, SR-33528 was evaluated at a lower concentration range (Fig 9B). These data reveal that SR-33528 potently inhibits ErbB4-dependent cell proliferation (IC_50_ = 1.05 nM) and IL3-dependent cell proliferation (IC_50_ = 2.51 nM). This minimal specificity for ErbB4 over IL3 indicates that this molecule may not be a prime candidate for further development as an ErbB4 inhibitor.

Due to the similarity in SR-33528 potency against ErbB4- and IL3-dependent proliferation, we postulated that SR-33528 targets a convergence point downstream of ErbB4 and the IL3 receptor (S5 Fig). Two such candidates are the PI3K/AKT signaling pathway and the Ras/Raf/MEK/ERK signaling pathway. However, SR-33528 fails to inhibit AKT phosphorylation or ERK phosphorylation. In light of these results, it is clear that SR-33528 does not act upstream of either AKT or ERK.

**Fig 10:**
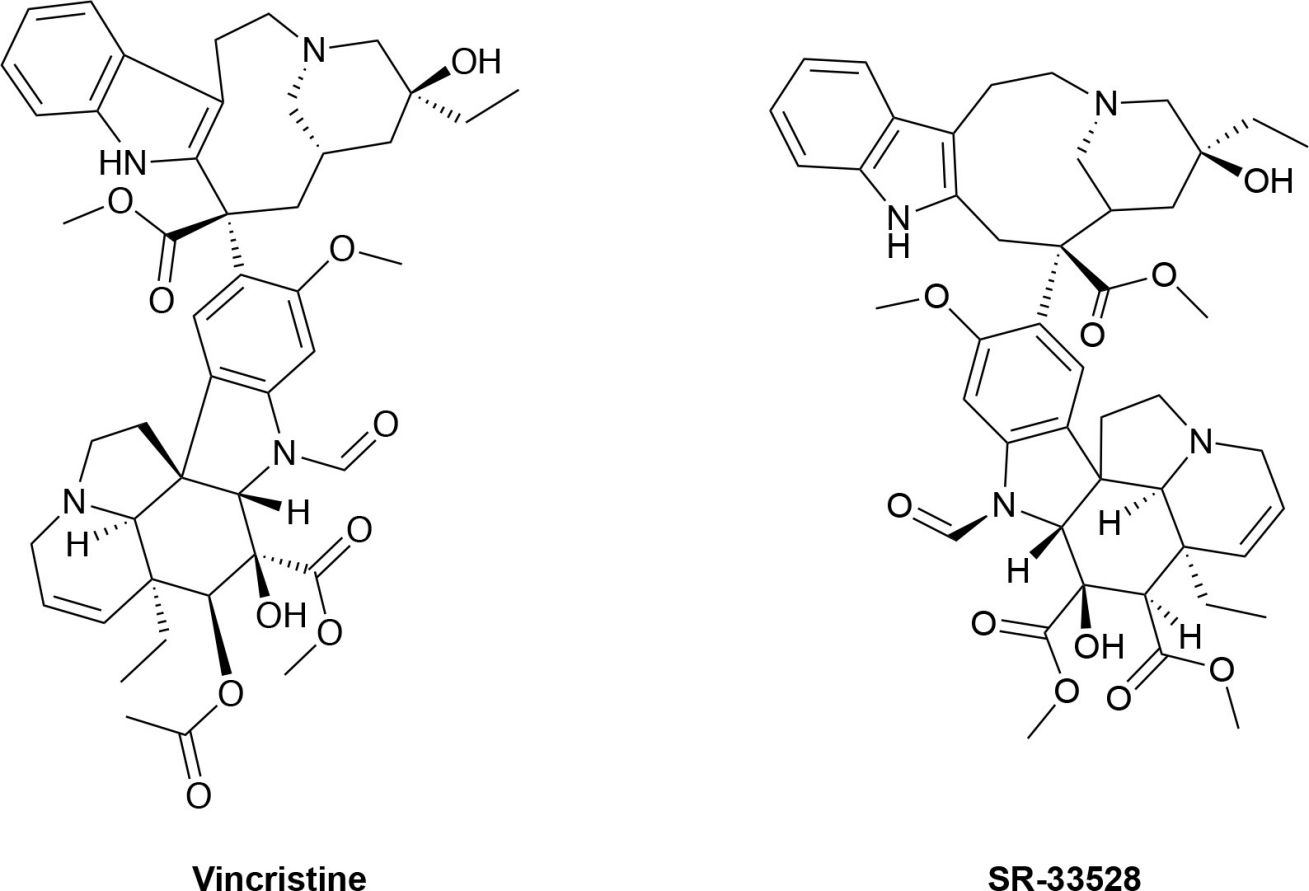
Chemical structure of vincristine and SR-33528.

In an attempt to identify the target for SR-33528, we performed an *in silico* search for molecules whose structure is similar to SR-33528. SR-33528 is structurally very similar to vinca alkaloids, particularly the FDA-approved anticancer agent vincristine (Fig 10) [25]. This similarity suggests that SR-33528, like vincristine, non-specifically inhibits cell proliferation by preventing tubulin polymerization into microtubules just prior to cell division. This mechanism of action is consistent with our observation that SR-33528 inhibits ErbB4- and IL3-dependent proliferation with roughly equivalent potency.

## 3. Discussion

In this report we have described the development and use of multiple screening strategies to identify molecles that stimulate ErbB4 tyrosine phosphorylation and inhibit ErbB4-dependent cell proliferation. As a result, six compounds (SR-33487, SR-33486, SR-33483, SR-33494, SR-33492, SR-33491) were identified that appear to selectively and potently inhibit ErbB4-dependent cell proliferation (Figs 6B and 8A–8E, Table 3).

Much work beyond the scope of what is described here is needed to advance the six compounds that appear to selectively and potently inhibit ErbB4-dependent cell proliferation. The mechanism(s) of action remains to be identified. Should these compounds function as *bona fide* direct inhibitors of ErbB4, traditional QSAR approaches will need to be deployed to improve the potency (and if necessary, selectivity) of these compounds. Of course, challenges of drug delivery, bioavailability, pharmacokinetics, and pharmacodynamics will need to be overcome. Nonetheless, these future efforts and challenges do not discount the importance of this work in establishing plausible, scalable approaches for identifying ErbB4 inhibitors.

Small molecule kinase inhibitors have been extensively studied as strategies for targeting tumors that are dependent on Epidermal Growth Factor Receptor (EGFR/ErbB1/HER1) or ErbB2 (HER2/Neu) signaling. However, the oncogenic activity of ErbB4 is dependent upon heterodimerization of ErbB4 with either EGFR or ErbB2, followed by phosphorylation of ErbB4 by EGFR or ErbB2. To be explicit, EGFR or ErbB2 kinase activity, but NOT ErbB4 kinase activity, is required for the oncogenic activity of EGFR-ErbB4 or ErbB2-ErbB4 heterodimers, respectively [7–13, 21]. Thus, small molecule ErbB4 tyrosine kinase inhibitors are not likely to be effective against tumors driven by the oncogenic activity of ErbB4 heterodimers.

Instead, our strategy for identifying small molecule ErbB4 inhibitors was inspired by our observation that the Q43L mutant of the ErbB4 ligand NRG2β is still able to stimulate ErbB4 tyrosine phosphorylation, but cannot stimulate the coupling of EGFR-ErbB4 heterodimers to cell proliferation. Moreover, the NRG2β/Q43L ErbB4 partial agonist inhibits the agonistic activity of wild-type NRG2β on EGFR-ErbB4 heterodimers [7, 26]. ErbB receptor partial agonists have already been translated to clinical practice, as the FDA-approved anti-ErbB2 monoclonal antibody transtuzumab stimulates ErbB2 tyrosine phosphorylation and downregulation, resulting in reduced ErbB2-dependent tumor cell proliferation [27]. Thus, using ErbB4 partial agonists to inhibit ErbB4-dependent tumor cell proliferation seems plausible. Finally, should the candidate ErbB4 partial agonists identified in this work fail to lead to drugs that can be used to treat ErbB4-dependent tumors, the screening strategies described here are also appropriate for screening anti-ErbB4 monoclonal antibody libraries for ErbB4 partial agonists. Indeed, given that the FDA-approved anti-ErbB receptor monoclonal antibodies trastuzumab, pertuzumab, and cetuximab each bind to a relatively large area of the target receptor, antibodies may be a better platform for developing ErbB4 partial agonists than small molecules.

## 4. Materials and methods

### 4.1. Cell lines, cell culture, recombinant NRGs, and inhibitors

The CEM/ErbB4 cells [17] were a gift from Dr. Gregory D. Plowman (Eli Lilly and Company, New York, New York, USA) through Dr. David F. Stern (Yale University, New Haven, Connecticut, USA). These cells were propagated using published culture conditions [9, 28]. The BaF3/EGFR+ErbB4 cells [8] were a gift from Dr. David F. Stern (Yale University, New Haven, Connecticut, USA) and were propagated under published culture conditions [8]. Standardized positive controls were included in experiments involving the CEM/ERBB4 and BaF3/EGFR+ERBB4 cell lines and results obtained using these positive controls were compared to historical results in order to monitor reproducibility and maintain authenticity. These cell lines were used no later than 10 passages after being thawed. These cell cultures were routinely tested for mycoplasma infection.

The U2OS cells (Catalog number 93-0465U3) were purchased from DiscoverX and maintained as instructed by DiscoverX. Experiments involving the U2OS cells utilized recently-thawed cells to maintain reproducibility by eliminating effects caused by selective evolution and by reducing the probability and consequence of mycoplasma infection.

Cell culture media and supplements were obtained from HyClone/Thermo Scientific, Gemini Bio-Products, DiscoverX, and Corning. Recombinant NRG1β was obtained from PeproTech (Catalog number 100–03, Lot number 0711316 and Lot number 0913316), and NRG2β isoforms and mutants have been described previously [9, 13, 26]. We have also previously described the procedures for expressing, purifying, and quantifying recombinant proteins [26]. The EGFR tyrosine kinase inhibitor gefitinib (Catalog number SC-202166) was acquired from Santa Cruz Biotechnology.

The library of small-molecule compounds (119,327 molecules) screened in this work was maintained by Southern Research (Birmingham, Alabama, USA). This library was assembled at Southern Research from purchased compounds obtained from multiple vendors. Although the compounds are non-proprietary, the composition of the library itself is not publically available.

### 4.2. DiscoverX pathHunter® U2OS ErbB4 functional assay

U2OS cells were cultured and the assay was performed according to the DiscoverX protocol [24]. On assay day one, 20 μL cells were added to each well of the plates at a concentration of 400,000 cells/mL for the final count of 8000 cells/well. The assay plates were incubated overnight at 37°C and 5% CO_2_ in a humidified atmosphere. On day two, compounds were diluted in assay medium (DiscoverX catalog number 93-0563R16B) to prepare a 5x concentrated dosing solution (50 μg/mL) and added to equilibrated assay plates in 5 μL (1/5 final well volume) to give a final compound concentration of 10 μg/mL and a final DMSO concentration of 0.2%. Assay medium alone (at 0.2% DMSO) served as the negative control and 100 nM NRG1β (at 0.2% DMSO) as the positive control. Drugging times were recorded, and plates were incubated at room temperature for 3 hours. Twelve (12) μL of detection reagent was added to the assay plates. Following 1h room-temperature incubation, ultrasensitive luminescence was read on a PerkinElmer EnVision plate reader.

### 4.3. Manual ligand stimulation and detection of ErbB4 tyrosine phosphorylation in CEM/ErbB4 cells

Ligand-induced ErbB4 tyrosine phosphorylation was stimulated using a published protocol [7]. Briefly, the CEM/ErbB4 cells were starved for 24 hours in serum- and factor-free base medium (RPMI) before being stimulated for 7 minutes on ice with an ErbB4 ligand (NRG1β, NRG2β, or NRG2β/Q43L) or candidate small molecule ErbB4 partial agonists. The cells were then lysed using a published protocol [7], and the lysates were immediately assayed for ErbB4 tyrosine phosphorylation or stored at -80°C until assayed.

ErbB4 tyrosine phosphorylation was analyzed using a 96-well “sandwich” enzyme-linked immunosorbent assay (ELISA) kit from R&D Systems (Catalog number DYC2115-2), which features an anti-ErbB4 capture antibody and an anti-phosphotyrosine antibody that is coupled to horseradish peroxidase (HRP). First, each well in a 96-well flat-bottom plate (ELISA plate) was coated with the anti-ErbB4 capture antibody, which recognizes the amino terminus of ErbB4. Nonspecific binding sites were then treated with a blocking buffer (1% BSA in PBS). Lysate samples containing ErbB4 were then added to the wells and incubated to permit ErbB4 binding to the immobilized anti-ErbB4 antibody. Then, an anti-phosphotyrosine antibody tagged with HRP was used to detect and visualize the phosphorylated tyrosine residues of the immobilized ErbB4 molecules. Oxidation of 3,3´,5,5´-tetramethylbenzidine by HRP (stopped using sulfuric acid) is indicative of ErbB4 tyrosine phosphorylation and was detected by measuring absorption at 450 nm. The data generated during the course of validating these assay conditions can be found in a (S1 Data).

### 4.4. Automated ligand stimulation and detection of ErbB4 tyrosine phosphorylation in CEM/ErbB4 cells

A Beckman Coulter Biomek 4000 automated liquid handling system was used to convert our standard, batch method for agonist stimulation of ErbB4 tyrosine phosphorylation [7] to a semi-automated 96-well methodology. (The liquid-handling script for this procedure using the Biomek 4000 is available upon request.) Briefly, the CEM/ErbB4 cells were starved for 24 hours in serum- and factor-free base medium (RPMI) before being stimulated in triplicate in 96-well conical-bottom plates (stimulation plates) for 7 minutes at 4°C and shaking with an ErbB4 ligand (NRG1β) or candidate small molecule ErbB4 partial agonists. After stimulation, lysis buffer was added to each well and incubated for 20 minutes at 4°C with shaking. Following incubation, the plates were centrifuged at 3200 RCF for 20 minutes at 4°C. The supernatants (lysate samples) were then immediately transferred to an ELISA plate and assayed for ErbB4 tyrosine phosphorylation or transfer to a storage plate and stored at -80°C until assayed.

Once again, a Biomek 4000 was used to convert the existing phospho-ErbB4 ELISA protocol to a semi-automated 96-well format. (The liquid-handling script for this procedure using the Biomek 4000 is available upon request.) Lysate samples were transferred from the 96-well stimulation plates to the 96-well ELISA plates that were precoated with the previously mentioned capture antibody. The phospho-ErbB4 ELISA was then completed as described previously using the Biomek 4000. The data obtained in the dose response experiments whose results are shown in Tables 1 and 2 can be found in a (S2 Data).

### 4.5. Automated ligand stimulation and detection of ErbB4 coupling to IL3 independence

We used an established manual 24-well assay [8] as the basis for developing a semi-automated 96-well assay for stimulation and detection of ErbB4-dependent cellular proliferation. (The liquid-handling script for this procedure using the Biomek 4000 is available upon request.) Briefly, BaF3/EGFR+ErbB4 cells were grown to saturation (2.5x10^6^ cells/mL) and aseptically seeded at 1x10^4^ cells/well (100 μL @ 1x10^5^ cells/mL) into a sterile 96-well microplate in the absence of IL3. The cells were then treated with an ErbB4 ligand (NRG1β) in triplicate. In studies of the inhibition of ErbB4-dependent cellular proliferation, the cells were treated with an ErbB4 ligand (NRG1β) in triplicate in the presence or absence of the EGFR tyrosine kinase inhibitor gefitinib or candidate small molecule inhibitors. The plate was incubated at 37°C and 5% CO_2_ for 120 hours before cell proliferation was analyzed using an MTT assay [20]. Briefly, cells were treated by directly adding 10 μL of MTT Reagent (3-(4,5-dimethylthiazol-2-yl)-2,5-diphenyltetrazolium bromide; ATCC^®^ 30-1010K™) to the culture medium in each well and incubated in the dark for 2 hours at 37°C and 5% CO_2_. Then, 100 μL of Detergent Reagent (ATCC^®^ 30-1010K™) was added to each well and the plate was incubated in the dark overnight at room temperature. MTT conversion was then determined by measuring the absorbance of each well at 570 nm. Dose response data were then analyzed using GraphPad Prism to determine the EC_50_ and E_max_ for NRG1β or the IC_50_ and I_max_ for inhibitors in the presence of NRG1β. As noted elsewhere and shown in S1 Fig, under these conditions MTT conversion is a function of differences in viable cell number, not a function of differences in cell metabolism. The data obtained in the dose response experiments whose results are shown in Table 3 and the associated figures can be found in a (S2 Data).

## 5. Conclusions

In this work, reliable, reproducible, and scalable approaches were developed, validated, and deployed for the discovery of compounds that hold potential as targeted cancer therapeutics. Our drug discovery approach was based on the observation that the Q43L mutant of the naturally occurring ErbB4 agonist NRG2β functions as a partial agonist of ErbB4. NRG2β/Q43L stimulates ErbB4 tyrosine phosphorylation, fails to stimulate ErbB4-dependent cell proliferation, and inhibits agonist-induced ErbB4-dependent cell proliferation. ErbB4 partial agonists hold promise as therapies for ErbB4-dependent tumors. Using the screening assays developed in this work, six compounds were identified that stimulate ErbB4 tyrosine phosphorylation, fail to stimulate ErbB4-dependent cell proliferation, and selectively inhibit ErbB4-dependent cell proliferation. While much work remains to further evaluate the therapeutic potential of these six compounds, this work has established a high-throughput screening process for the identification of ErbB4 inhibitors.

## Supporting information

S1 Fig MTT conversion by BaF3/EGFR+ErbB4 cells correlates with cell number. BaF3/EGFR+ErbB4 cells were stimulated with increasing concentrations of NRG1β as depicted and essentially as described in section 4.5. MTT conversion was assayed essentially as described in section 4.5. In parallel wells, dead BaF3/EGFR+ErbB4 cells were stained with trypan blue and viable cells were counted using a hemocytometer. MTT conversion and cell number were plotted as a function of NRG1β concentration. The apparent NRG1β EC_50_ (with respect to MTT conversion) in this experiment is different from that reported in Fig 2A. This is due to assay optimization that was performed after this experiment.
(TIF)

S2 Fig Four candidates do not selectively and potently inhibit ErbB4-dependent cellular proliferation. (a-d) In three independent trials and using a modified version of our semi-automated processes, BaF3/EGFR+ErbB4 cells were treated with increasing concentrations of each candidate inhibitor in the presence of 0.1 nM IL3 or 0.3 nM NRG1β. A semi-automated MTT assay was used to analyze cellular proliferation 120 hours post-stimulation. Curves were fit to the data using GraphPad Prism to determine the IC_50_ value for each candidate against 0.1 nM IL3 and 0.3 nM NRG1β. IC_50_ values are also shown in Table 3.
(TIF)

S3 Fig Four candidates do not selectively and potently inhibit ErbB4-dependent cellular proliferation. (a-d) In three independent trials and using a modified version of our semi-automated processes, BaF3/EGFR+ErbB4 cells were treated with increasing concentrations of each candidate inhibitor in the presence of 0.1 nM IL3 or 0.3 nM NRG1β. A semi-automated MTT assay was used to analyze cellular proliferation 120 hours post-stimulation. Curves were fit to the data using GraphPad Prism to determine the IC_50_ value for each candidate against 0.1 nM IL3 and 0.3 nM NRG1β. IC_50_ values are also shown in Table 3.
(TIF)

S4 Fig Four candidates are no longer under consideration. (a-d) In three independent trials and using a modified version of our semi-automated processes, BaF3/EGFR+ErbB4 cells were treated with increasing concentrations of each candidate inhibitor in the presence of 0.1 nM IL3 or 0.3 nM NRG1β. A semi-automated MTT assay was used to analyze cellular proliferation 120 hours post-stimulation. Curves were fit to the data using GraphPad Prism to determine the IC_50_ value for each candidate against 0.1 nM IL3 and 0.3 nM NRG1β. IC_50_ values are also shown in Table 3.
(TIF)

S5 Fig Depiction of ErbB4 and IL3 receptor signaling pathways. (TIF)

S1 Data Raw data generated during the course of validating the anti-phospho-ErbB4 ELISA assay conditions. The file was generated using Microsoft Excel and the file is in the Microsoft Excel.xlsx format.
(XLSX)

S2 Data Raw data generated by the analyses of ErbB4 tyrosine phosphorylation and by the analyses of ErbB4 coupling to IL3 independence. The file was generated using GraphPad Prism version 6 and the file is in the GraphPad Prism.pzfx format.
(PZFX)

## References

[1] Kuske M, Westphal D, Wehner R, Schmitz M, Beissert S, Praetorius C, et al Immunomodulatory effects of BRAF and MEK inhibitors: Implications for Melanoma therapy. Pharmacol Res. 2018;136:151–9. 10.1016/j.phrs.2018.08.019 . 30145328

[2] Long GV, Eroglu Z, Infante J, Patel S, Daud A, Johnson DB, et al Long-Term Outcomes in Patients With BRAF V600-Mutant Metastatic Melanoma Who Received Dabrafenib Combined With Trametinib. J Clin Oncol. 2018;36(7):667–73. 10.1200/JCO.2017.74.1025 . 28991513 PMC10466457

[3] Long GV, Grob JJ, Nathan P, Ribas A, Robert C, Schadendorf D, et al Factors predictive of response, disease progression, and overall survival after dabrafenib and trametinib combination treatment: a pooled analysis of individual patient data from randomised trials. Lancet Oncol. 2016;17(12):1743–54. 10.1016/S1470-2045(16)30578-2 . 27864013

[4] Hamid O, Robert C, Daud A, Hodi FS, Hwu WJ, Kefford R, et al Five-year survival outcomes for patients with advanced melanoma treated with pembrolizumab in KEYNOTE-001. Ann Oncol. 2019 10.1093/annonc/mdz011 . 30715153 PMC6503622

[5] Robert C, Schachter J, Long GV, Arance A, Grob JJ, Mortier L, et al Pembrolizumab versus Ipilimumab in Advanced Melanoma. N Engl J Med. 2015;372(26):2521–32. 10.1056/NEJMoa1503093 . 25891173

[6] Wolchok JD, Chiarion-Sileni V, Gonzalez R, Rutkowski P, Grob JJ, Cowey CL, et al Overall Survival with Combined Nivolumab and Ipilimumab in Advanced Melanoma. N Engl J Med. 2017;377(14):1345–56. 10.1056/NEJMoa1709684 28889792 PMC5706778

[7] Wilson KJ, Mill CP, Gallo RM, Cameron EM, VanBrocklin H, Settleman J, et al The Q43L mutant of neuregulin 2beta is a pan-ErbB receptor antagonist. Biochem J. 2012;443(1):133–44. 10.1042/BJ20110921 22216880 PMC3960720

[8] Riese DJ 2nd, van Raaij TM, Plowman GD, Andrews GC, Stern DF. The cellular response to neuregulins is governed by complex interactions of the erbB receptor family. Mol Cell Biol. 1995;15(10):5770–6. 10.1128/mcb.15.10.5770 7565730 PMC230829

[9] Hobbs SS, Coffing SL, Le AT, Cameron EM, Williams EE, Andrew M, et al Neuregulin isoforms exhibit distinct patterns of ErbB family receptor activation. Oncogene. 2002;21(55):8442–52. 10.1038/sj.onc.1205960 . 12466964

[10] Riese DJ 2nd, Bermingham Y, van Raaij TM, Buckley S, Plowman GD, Stern DF. Betacellulin activates the epidermal growth factor receptor and erbB-4, and induces cellular response patterns distinct from those stimulated by epidermal growth factor or neuregulin-beta. Oncogene. 1996;12(2):345–53. . 8570211

[11] Riese DJ, Kim ED, Elenius K, Buckley S, Klagsbrun M, Plowman GD, et al The epidermal growth factor receptor couples transforming growth factor-alpha, heparin-binding epidermal growth factor-like factor, and amphiregulin to Neu, ErbB-3, and ErbB-4. J Biol Chem. 1996;271(33):20047–52. 10.1074/jbc.271.33.20047 . 8702723

[12] Riese DJ 2nd, Komurasaki T, Plowman GD, Stern DF. Activation of ErbB4 by the bifunctional epidermal growth factor family hormone epiregulin is regulated by ErbB2. J Biol Chem. 1998;273(18):11288–94. 10.1074/jbc.273.18.11288 . 9556621

[13] Hobbs SS, Cameron EM, Hammer RP, Le AT, Gallo RM, Blommel EN, et al Five carboxyl-terminal residues of neuregulin2 are critical for stimulation of signaling by the ErbB4 receptor tyrosine kinase. Oncogene. 2004;23(4):883–93. 10.1038/sj.onc.1207250 . 14661053

[14] Wu P, Nielsen TE, Clausen MH. FDA-approved small-molecule kinase inhibitors. Trends Pharmacol Sci. 2015;36(7):422–39. 10.1016/j.tips.2015.04.005 . 25975227

[15] Arteaga CL, Engelman JA. ERBB receptors: from oncogene discovery to basic science to mechanism-based cancer therapeutics. Cancer Cell. 2014;25(3):282–303. 10.1016/j.ccr.2014.02.025 24651011 PMC4018830

[16] Hynes NE, Lane HA. ERBB receptors and cancer: the complexity of targeted inhibitors. Nat Rev Cancer. 2005;5(5):341–54. 10.1038/nrc1609 . 15864276

[17] Plowman GD, Green JM, Culouscou JM, Carlton GW, Rothwell VM, Buckley S. Heregulin induces tyrosine phosphorylation of HER4/p180erbB4. Nature. 1993;366(6454):473–5. 10.1038/366473a0 . 7902537

[18] Engvall E, Perlmann P. Enzyme-linked immunosorbent assay (ELISA). Quantitative assay of immunoglobulin G. Immunochemistry. 1971;8(9):871–4. 10.1016/0019-2791(71)90454-x . 5135623

[19] Zhang JH, Chung TD, Oldenburg KR. A Simple Statistical Parameter for Use in Evaluation and Validation of High Throughput Screening Assays. J Biomol Screen. 1999;4(2):67–73. 10.1177/108705719900400206 . 10838414

[20] Mosmann T. Rapid colorimetric assay for cellular growth and survival: application to proliferation and cytotoxicity assays. J Immunol Methods. 1983;65(1–2):55–63. 10.1016/0022-1759(83)90303-4 . 6606682

[21] Mill CP, Zordan MD, Rothenberg SM, Settleman J, Leary JF, Riese DJ 2nd. ErbB2 Is Necessary for ErbB4 Ligands to Stimulate Oncogenic Activities in Models of Human Breast Cancer. Genes Cancer. 2011;2(8):792–804. Epub 2012/03/07. 10.1177/1947601911431080 22393464 PMC3278901

[22] Hennequin LF, Ballard P, Boyle FT, Delouvrie B, Ellston RP, Halsall CT, et al Novel 4-anilinoquinazolines with C-6 carbon-linked side chains: synthesis and structure-activity relationship of a series of potent, orally active, EGF receptor tyrosine kinase inhibitors. Bioorg Med Chem Lett. 2006;16(10):2672–6. 10.1016/j.bmcl.2006.02.025 . 16516473

[23] Wakeling AE, Guy SP, Woodburn JR, Ashton SE, Curry BJ, Barker AJ, et al ZD1839 (Iressa): an orally active inhibitor of epidermal growth factor signaling with potential for cancer therapy. Cancer Res. 2002;62(20):5749–54. . 12384534

[24] PathHunter® U2OS ErbB4 Functional Assay []. [cited 2018 Jan 24]. Available from: https://www.discoverx.com/products/cell-line/u2os-erbb4-functional-assay-93-0465c3.

[25] Rowinsky E. The Vinca Alkaloids Holland-Frei Cancer Medicine 6th edition Hamilton (ON): BC Decker; 2003.

[26] Wilson KJ, Mill CP, Cameron EM, Hobbs SS, Hammer RP, Riese DJ 2nd. Inter-conversion of neuregulin2 full and partial agonists for ErbB4. Biochem Biophys Res Commun. 2007;364(2):351–7. 10.1016/j.bbrc.2007.10.004 17945187 PMC2094732

[27] Riese DJ 2nd. Ligand-based receptor tyrosine kinase partial agonists: New paradigm for cancer drug discovery? Expert Opin Drug Discov. 2011;6(2):185–93. Epub 2011/05/03. 10.1517/17460441.2011.547468 21532939 PMC3083243

[28] Sweeney C, Lai C, Riese DJ 2nd, Diamonti AJ, Cantley LC, Carraway KL 3rd. Ligand discrimination in signaling through an ErbB4 receptor homodimer. J Biol Chem. 2000;275(26):19803–7. Epub 2000/06/27. 10.1074/jbc.C901015199 . 10867024

