# Supplementary figures and images for: Development and application of high-throughput screens for the discovery of compounds that disrupt ErbB4 signaling: Candidate cancer therapeutics
Source: PLoS One. 2020 Dec 30;15(12):e0243901. doi: 10.1371/journal.pone.0243901 (PMC7773179; doi:10.1371/journal.pone.0243901)

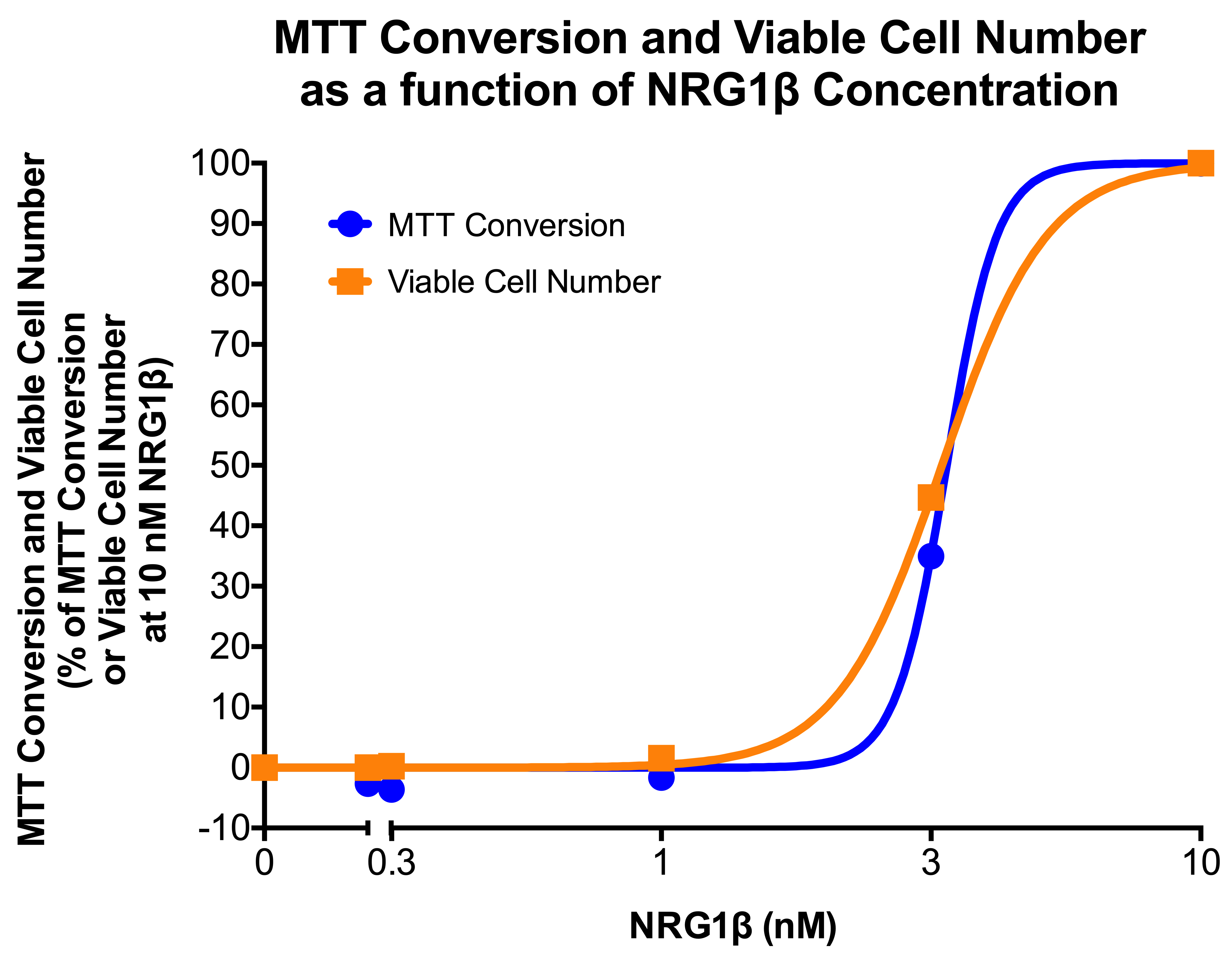

Supplement: S1 Fig — BaF3/EGFR+ErbB4 cells were stimulated with increasing concentrations of NRG1β as depicted and essentially as described in section 4.5. MTT conversion was assayed essentially as described in section 4.5. In parallel wells, dead BaF3/EGFR+ErbB4 cells were stained with trypan blue and viable cells were counted using a hemocytometer. MTT conversion and cell number were plotted as a function of NRG1β concentration. The apparent NRG1β EC50 (with respect to MTT conversion) in this experiment is different from that reported in Fig 2A. This is due to assay optimization that was performed after this experiment. (TIF) [file pone.0243901.s001.tif]

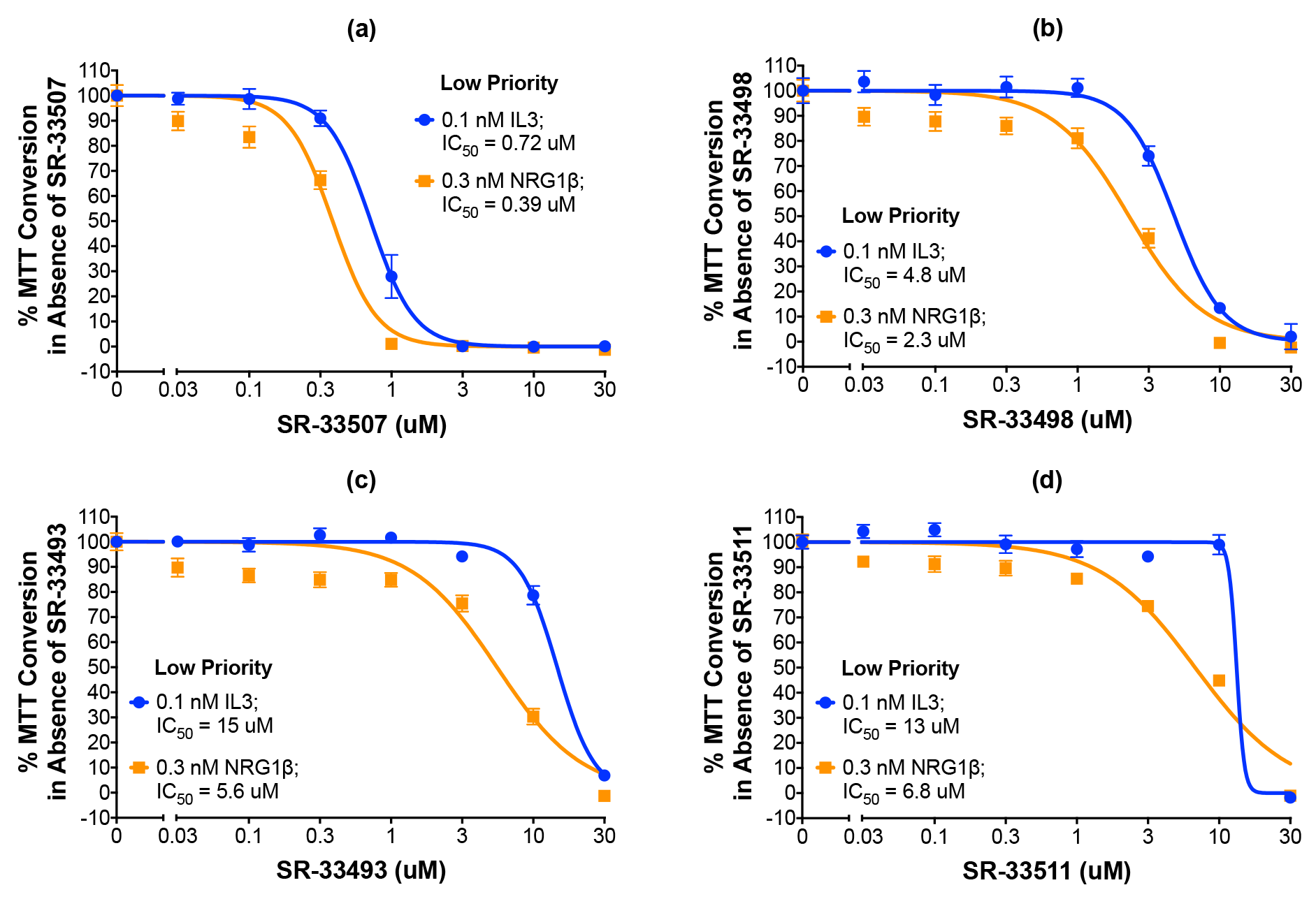

Supplement: S2 Fig — (a-d) In three independent trials and using a modified version of our semi-automated processes, BaF3/EGFR+ErbB4 cells were treated with increasing concentrations of each candidate inhibitor in the presence of 0.1 nM IL3 or 0.3 nM NRG1β. A semi-automated MTT assay was used to analyze cellular proliferation 120 hours post-stimulation. Curves were fit to the data using GraphPad Prism to determine the IC50 value for each candidate against 0.1 nM IL3 and 0.3 nM NRG1β. IC50 values are also shown in Table 3. (TIF) [file pone.0243901.s002.tif]

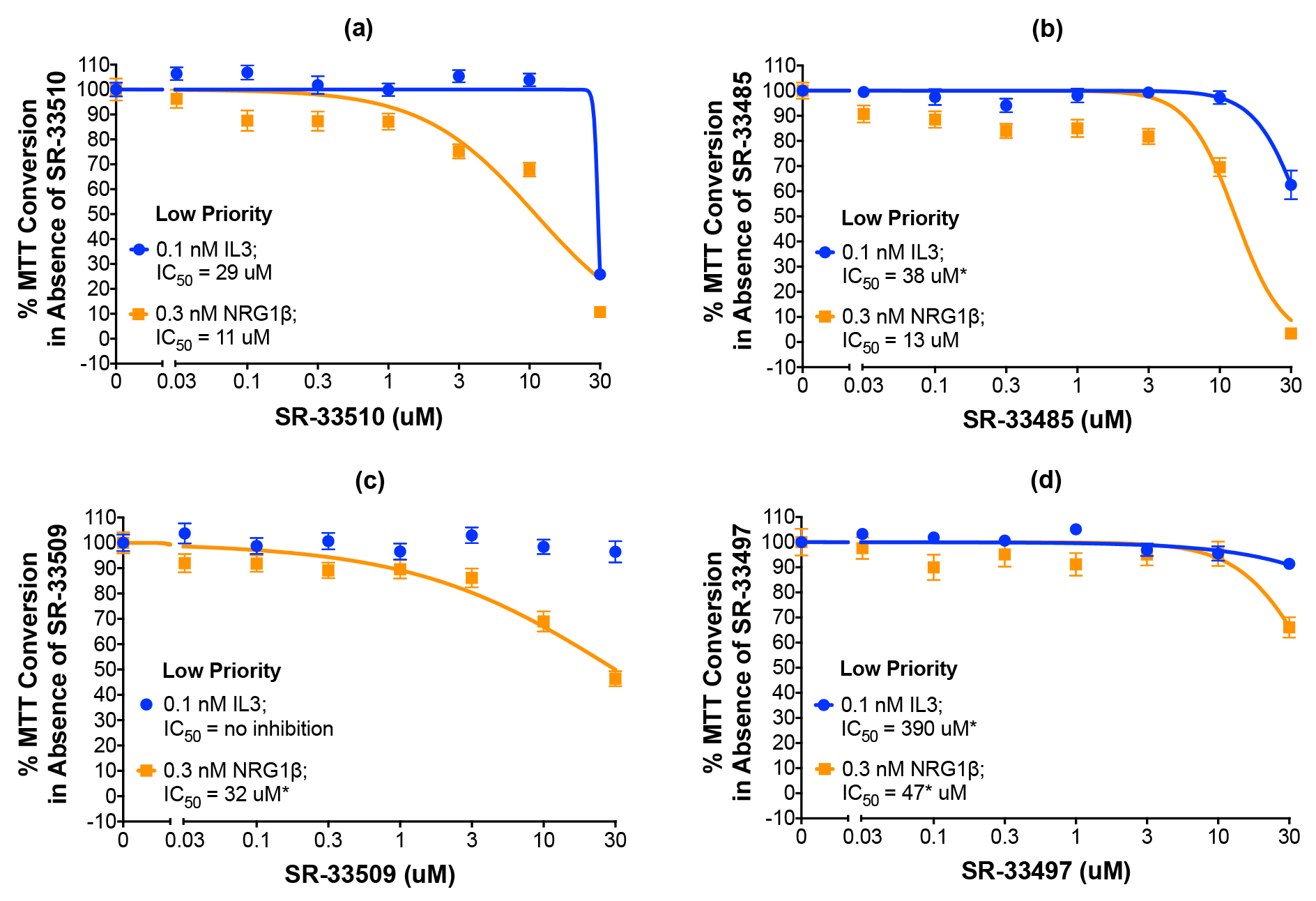

Supplement: S3 Fig — (a-d) In three independent trials and using a modified version of our semi-automated processes, BaF3/EGFR+ErbB4 cells were treated with increasing concentrations of each candidate inhibitor in the presence of 0.1 nM IL3 or 0.3 nM NRG1β. A semi-automated MTT assay was used to analyze cellular proliferation 120 hours post-stimulation. Curves were fit to the data using GraphPad Prism to determine the IC50 value for each candidate against 0.1 nM IL3 and 0.3 nM NRG1β. IC50 values are also shown in Table 3. (TIF) [file pone.0243901.s003.tif]

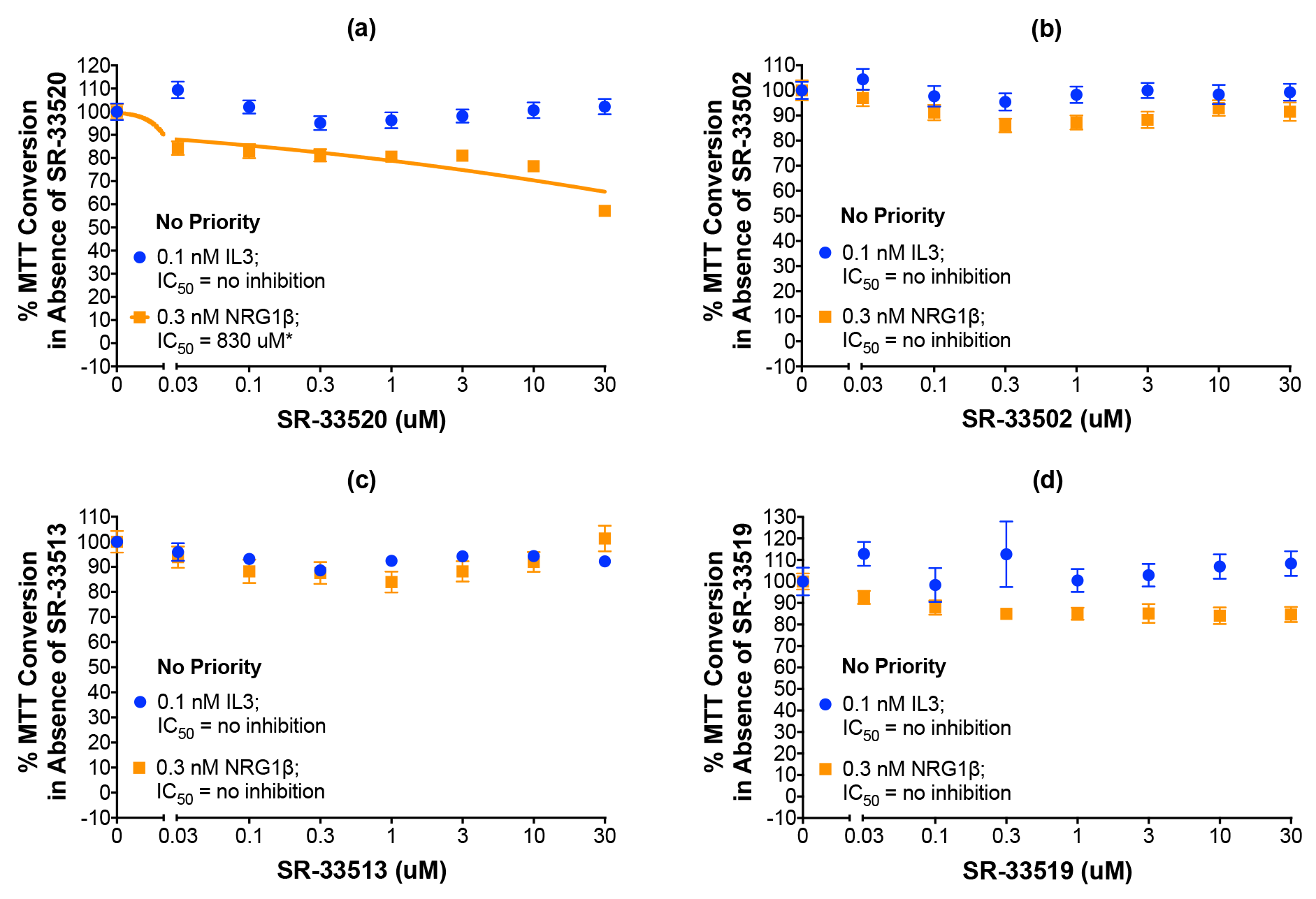

Supplement: S4 Fig — (a-d) In three independent trials and using a modified version of our semi-automated processes, BaF3/EGFR+ErbB4 cells were treated with increasing concentrations of each candidate inhibitor in the presence of 0.1 nM IL3 or 0.3 nM NRG1β. A semi-automated MTT assay was used to analyze cellular proliferation 120 hours post-stimulation. Curves were fit to the data using GraphPad Prism to determine the IC50 value for each candidate against 0.1 nM IL3 and 0.3 nM NRG1β. IC50 values are also shown in Table 3. (TIF) [file pone.0243901.s004.tif]

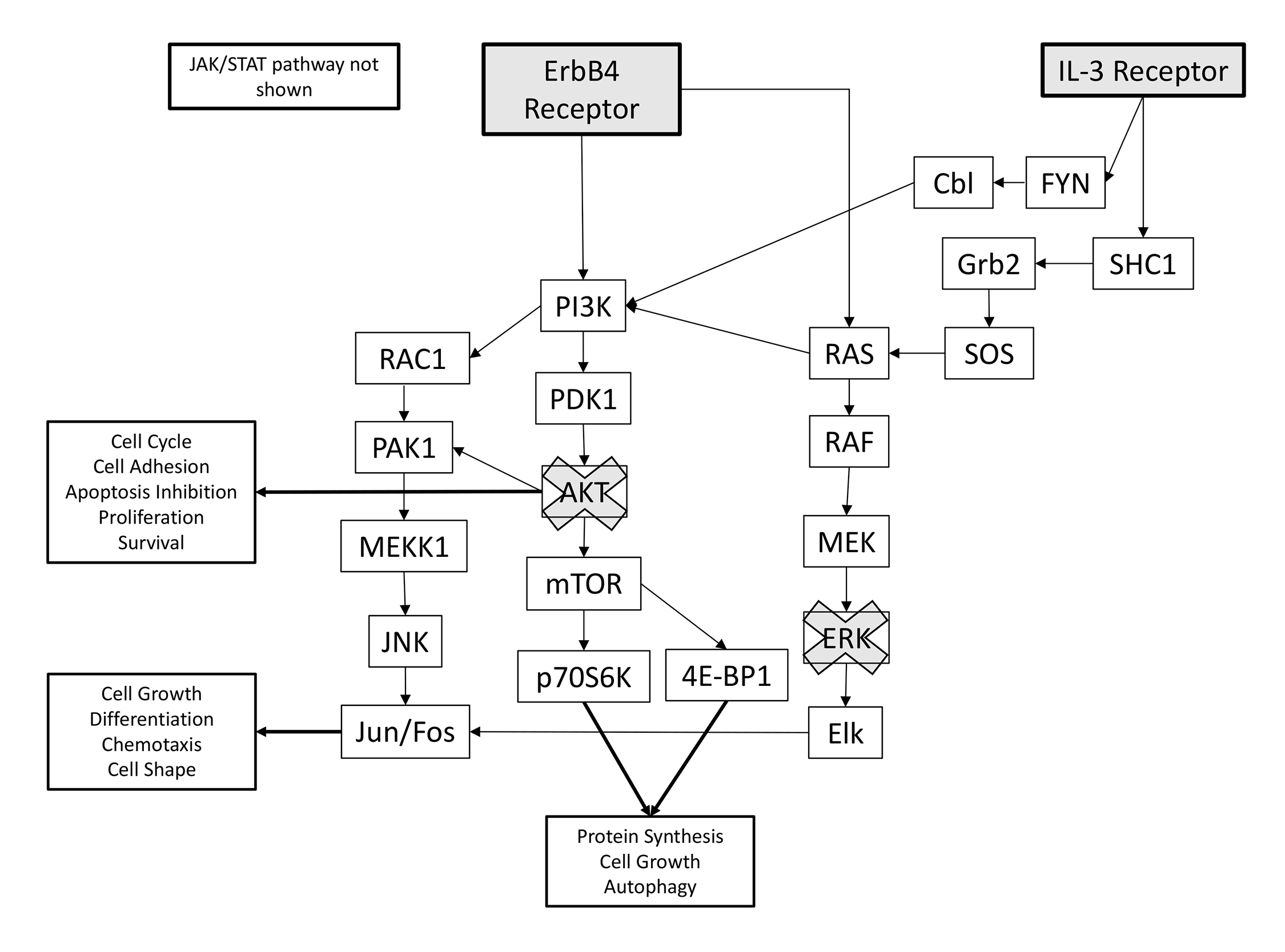

Supplement: S5 Fig — (TIF) [file pone.0243901.s005.tif]
